# Advanced Echocardiographic Characterization of Neonatal Ebstein’s Anomaly Using Myocardial Deformation Imaging: A Single-Center Study

**DOI:** 10.3390/life16040670

**Published:** 2026-04-14

**Authors:** Carmen Corina Șuteu, Nicola Şuteu, Liliana Gozar, Oana Cristina Marginean, Andreea Cerghit-Paler, Maria Oana Săsăran, Camelia Râtea, Amalia Făgărăşan

**Affiliations:** 1 Department of Pediatrics III, “George Emil Palade” University of Medicine, Pharmacy, Science, and Technology of Târgu Mureș, 540136 Târgu Mureș, Romania; suteucarmen@yahoo.com (C.C.Ș.); lili_gozar@yahoo.com (L.G.); amalia_fagarasan@yahoo.com (A.F.); 2 Department of Pediatric Cardiology, Emergency Institute for Cardiovascular Diseases and Transplantation, 540136 Târgu Mureş, Romania; 3 Department of Pediatrics I, “George Emil Palade” University of Medicine, Pharmacy, Science, and Technology of Târgu Mureș, 540136 Târgu Mureş, Romania; oana.marginean@umfst.ro; 4 Department of Pediatrics III, Faculty of Medicine English, “George Emil Palade” University of Medicine, Pharmacy, Science, and Technology of Târgu Mureș, 540136 Târgu Mureş, Romania; palerandreea@yahoo.com (A.C.-P.); oanam93@yahoo.com (M.O.S.); 5 Moldova & ProNutrition Center, Doctoral School, “Nicolae Testemitanu” State University of Medicine and Pharmacy of the Republic of Moldova, MD-2004 Chişinău, Moldova; camelia.ratea@mitogenix.ro

**Keywords:** Ebstein’s anomaly, neonates, speckle-tracking echocardiography, myocardial strain, atrial strain

## Abstract

Background: Neonatal Ebstein’s anomaly (EA) is a severe condition with significant hemodynamic instability and early myocardial dysfunction, where abnormal right-heart geometry limits conventional echocardiography and highlights the value of myocardial deformation imaging. Methods: We conducted a single-center retrospective observational study including 16 neonates with EA and 26 healthy neonates. All subjects underwent comprehensive transthoracic echocardiography during the neonatal period. Conventional two-dimensional imaging and speckle-tracking echocardiography (STE) were used to assess biventricular and biatrial myocardial deformation. Deformation parameters were compared between groups, and receiver operating characteristic (ROC) curve analysis evaluated diagnostic performance. Results: Neonates with EA demonstrated significant structural remodeling and severe biventricular and biatrial dysfunction compared with controls. Speckle-tracking showed markedly reduced right ventricular longitudinal strain (LS) in all segments (all, *p* < 0.001), particularly in free-wall and four-chamber views. Left ventricular (LV) global LS (GLS) was significantly reduced in neonates with EA compared with controls (−14.53% vs. −22.32%, *p* < 0.001), indicating early involvement of LV myocardial function in the neonatal period. Atrial reservoir, conduit, and contractile strain were severely impaired in both atria (all, *p* < 0.001). ROC analysis revealed excellent diagnostic accuracy, especially for LVGLS (AUC 0.919) and right atrial contractile strain (AUC 0.958). Conclusions: STE enables the early detection of extensive biventricular and biatrial myocardial dysfunction in neonatal EA, including abnormalities not fully captured by conventional echocardiographic parameters, thereby providing significant incremental diagnostic value.

## 1. Introduction

Ebstein’s anomaly (EA) is a rare congenital malformation of the tricuspid valve (TV), accounting for <1% of all congenital heart defects and occurring in approximately 1 per 20,000 live births [1]. The primary embryologic defect is incomplete delamination of the septal and posterior leaflets from the right ventricular (RV) myocardium, resulting in apical displacement of the leaflet hinge points and a downward migration of the functional tricuspid annulus [1]. This abnormal development creates a variable “atrialized” RV (aRV) segment, thinning of the atrialized myocardial wall, reduction of the functional RV (FRV), and often severe tricuspid regurgitation (TR) [2]. The anterior leaflet of TV frequently exhibits additional deformities, including redundancy, elongation, fenestration, or tethering to the RV free wall, which further contribute to valvular insufficiency and right-sided volume overload [2,3].

Neonatal presentation of EA reflects the most severe end of the pathological spectrum and is frequently associated with cyanosis and right-sided heart failure, with perinatal mortality approaching 45% in critical forms [1]. Elevated pulmonary vascular resistance (PVR) in the early neonatal period exacerbates the hemodynamic burden of severe TR, generating marked right atrial (RA) dilation and right-to-left shunting across an interatrial communication, leading to systemic desaturation [2,4]. In advanced anatomical forms (Carpentier types C-D), the markedly reduced FRV frequently cannot provide antegrade pulmonary blood flow, producing a physiology that resembles pulmonary atresia [1]. The massively dilated right-sided chambers may further compress the left ventricle (LV), resulting in the characteristic “pancaked” LV configuration and compromised systemic output [2,5]. In extreme forms, a hemodynamic “circular shunt” may occur, wherein ductal flow recirculates through the pulmonary artery, across the incompetent TV, and returns to the left atrium (LA), effectively bypassing pulmonary gas exchange and causing profound cyanosis [1]. Additionally, severe fetal cardiomegaly may impair lung development and result in varying degrees of pulmonary hypoplasia, further worsening postnatal outcomes [1,2].

Importantly, EA is increasingly recognized not only as a valvular malformation but also as a primary RV myocardial disease. Histopathological findings demonstrate reduced myocardial fiber density, fibrosis, and intrinsic contractile abnormalities within both the aRV and FRV segments, providing a mechanistic basis for RV dysfunction even when TR is not severe [1]. These myocardial abnormalities hold significant diagnostic and prognostic implications, particularly in the neonatal period.

Accurate hemodynamic and anatomical assessment of the RV is essential for early risk stratification. Conventional echocardiographic parameters, including tricuspid annular plane systolic excursion (TAPSE), tissue Doppler-derived S velocity, and RV fractional area change (RV_FAC), are widely used to evaluate RV systolic function. However, these indices are highly load-dependent and often unreliable in EA, owing to cardiac distortion, abnormal RV geometry, and highly variable loading conditions, particularly in neonates [4]. Thus, advanced imaging modalities have become increasingly relevant. Real-time three-dimensional (3D) echocardiography provides detailed visualization of leaflet morphology, commissures, and coaptation defects, improving surgical planning in complex anatomy [3].

Speckle-tracking echocardiography (STE), by quantifying myocardial deformation, offers a more sensitive and relatively load-independent method for assessing RV and RA function. STE has demonstrated prognostic value in both pediatric and adult EA populations. Reductions in global RV longitudinal strain and RA strain correlate strongly with disease severity and predict adverse outcomes such as arrhythmia, heart failure, or the need for surgery [5]. In pediatric EA, RV longitudinal strain and RA strain have been shown to independently predict clinical deterioration during long-term follow-up [6].

In neonatal EA, marked dilation and geometric distortion of right-sided chambers adversely affect LV filling and systolic performance, highlighting the necessity for a biventricular approach to functional assessment. Although the primary pathology involves the right heart, LV dysfunction constitutes an important determinant of disease severity in neonates and is largely mediated by ventricular interdependence, interventricular septal displacement, and mechanical compression from enlarged right-sided chambers, despite preserved intrinsic LV morphology [2,5]. Conventional echocardiographic indices may underestimate this dysfunction. Speckle-tracking echocardiography enables sensitive detection of subclinical LV systolic impairment, with reduced LV global longitudinal strain (GLS) correlating with disease severity and adverse ventricular interaction in pediatric and adult EA populations [5,6]. These findings underscore the importance of comprehensive biventricular deformation analysis for early risk stratification, including in the neonatal period [7].

Despite these advances, investigations specifically focused on the neonatal population remain limited. Given the unique transitional physiology and heightened vulnerability of neonates with EA, including the risk of hemodynamic collapse, severe hypoxemia, and early mortality, there is a critical need to improve early functional assessment. Advanced imaging, particularly STE, may offer a more precise understanding of RV and RA dysfunction in this high-risk cohort, supporting timely and more personalized clinical decision-making.

Therefore, the aim of this study is to evaluate whether STE can detect early biventricular and biatrial myocardial dysfunction in neonates with EA, including abnormalities not fully captured by conventional echocardiographic assessment, thereby providing incremental diagnostic value.

## 2. Materials and Methods

### 2.1. Study Design and Population

This observational, single-center study enrolled neonates with a confirmed diagnosis of EA who were admitted to the Pediatric Cardiology Department of the Emergency Institute for Cardiovascular Diseases and Transplantation, Târgu Mureș, Romania, a national tertiary referral center for pediatric cardiac care, between January 2020 and January 2025. Sixteen neonates with EA met the inclusion criteria. Exclusion criteria comprised associated major congenital heart defects that could independently affect RV geometry or TV morphology, including anatomical pulmonary atresia or critical pulmonary stenosis, tetralogy of Fallot, and complete atrioventricular canal defects.

A control group consisted of 26 healthy term neonates with no structural or functional cardiac abnormalities, evaluated in our department for an innocent systolic murmur. Only neonates with complete, high-quality echocardiographic recordings available in the institutional imaging archive were eligible for inclusion.

The study was conducted in accordance with the Declaration of Helsinki, and approved by the Ethics Committee of George Emil Palade University of Medicine, Pharmacy, Science, and Technology of Târgu-Mureș, No 3589 from 29 January 2025. Written informed consent was obtained from the legal guardians of all participants.

### 2.2. Clinical Data Collection

Clinical data were retrospectively extracted from medical records and included demographic characteristics, anthropometric measurements, peripheral oxygen saturation (SaO_2_), blood pressure (BP), heart rate (HR), anatomical features, and relevant paraclinical findings related to EA physiology. The cardio-thoracic (CT) index was assessed using chest radiographs obtained within the same hospitalization period.

### 2.3. Echocardiography

**Figure 1:**
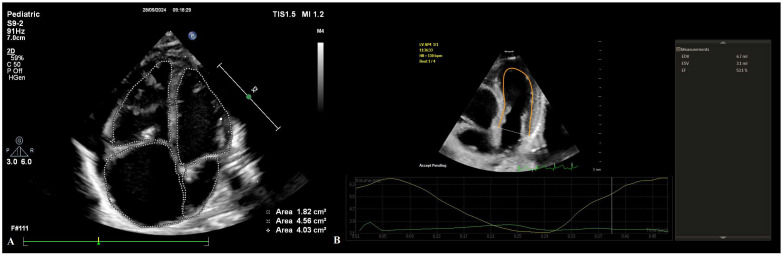
Representative conventional two-dimensional echocardiographic assessment in a neonate with Ebstein’s anomaly. Legend: (**A**) Apical four-chamber view illustrating the calculation of the Great Ormond Street Echocardiographic (GOSE) score, defined as the ratio of the combined area of the right atrium and atrialized right ventricle to the area of the functional right ventricle, left ventricle, and left atrium; (**B**) Left ventricular ejection fraction (LV_EF) measurement using Simpson’s biplane method.

**Figure 2:**
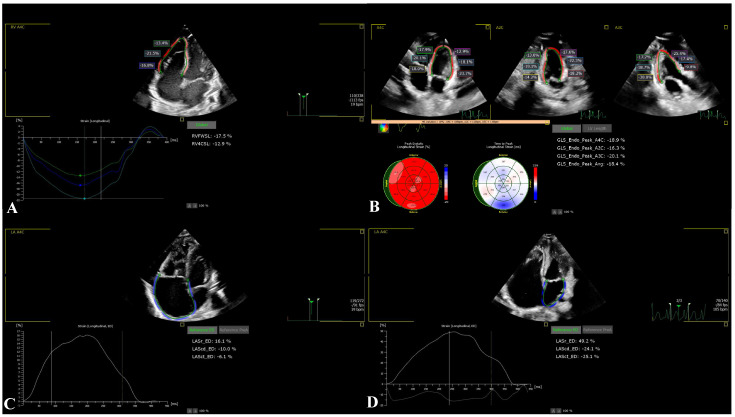
Representative speckle-tracking echocardiography (STE) analysis in a neonate with Ebstein’s anomaly. Legend: (**A**) Right ventricular longitudinal strain analysis from the apical four-chamber view, including free-wall and four-chamber strain curves; (**B**) Left ventricular global longitudinal strain (LV_GLS) derived from apical four-, two-, and three-chamber views, with corresponding bull’s-eye plot; (**C**) Left atrial strain analysis demonstrating reservoir, conduit, and contractile phases; (**D**) Right atrial strain analysis demonstrating impaired atrial deformation.

All enrolled neonates underwent comprehensive transthoracic echocardiographic evaluation immediately after birth using a Philips EPIQ CVx ultrasound system (Philips Healthcare, Andover, MA, USA) equipped with an X5-1 matrix-array transducer. Speckle-tracking echocardiography analysis was performed using dedicated software (Philips QLab, version 15.0; material number 453562090801). Echocardiographic examinations were conducted in accordance with the recommendations of the American Society of Echocardiography (ASE) for chamber quantification and myocardial deformation imaging [8]. All image acquisitions and measurements were performed by a single investigator with more than five years of experience in neonatal transthoracic echocardiography and STE analysis. Image quality was rigorously evaluated prior to inclusion; only loops with optimal visualization of endocardial borders across all myocardial segments were accepted. Studies with incomplete endocardial delineation, relevant artifacts, or inadequate frame rates were excluded to ensure measurement reliability. Representative examples of conventional and speckle-tracking echocardiographic analysis are shown in Figure 1 and Figure 2.

#### 2.3.1. Conventional 2D Echocardiographic Examination

Following an initial anatomical survey, conventional 2D echocardiographic measurements were obtained in accordance with ASE guidelines [8]. The anatomical assessment included detailed evaluation of TV morphology, annular diameter with corresponding z-scores, leaflet mobility, and the degree of apical displacement. Tricuspid regurgitation (TR) severity was assessed using color Doppler imaging. Pulmonary valve (PV) morphology, annular diameter with z-scores, leaflet motion, RV forward flow (RVFF), and the presence and severity of pulmonary regurgitation were also evaluated. Cardiac chamber quantification included measurements of the FRV, aRV, RA, LA, and LV areas. Derived indices included the Great Ormond Street Echocardiographic (GOSE) score (the ratio of the combined area of the RA and aRV to the area of the FRV, LA, and LV), RV index (FRV area/body surface area), LV index (LV area/body surface area), and RA index (RA area/body surface area) (Figure 1A).

Functional assessment comprised established parameters of RV and LV systolic and diastolic performance. Systolic function was evaluated using TAPSE, RV_FAC, lateral mitral annular plane systolic excursion (MAPSE), and LV ejection fraction (LV_EF). LV_EF was calculated using Simpson’s biplane method, with end-diastole defined as the frame corresponding to maximal LV volume and end-systole as the frame with minimal LV volume (Figure 1B). Tissue Doppler-derived systolic (S) velocities of the tricuspid and mitral annuli were also obtained. Diastolic function was assessed using pulsed-wave Doppler analysis of mitral and tricuspid inflow velocities (E/A ratio), complemented by tissue Doppler imaging of the lateral annuli to obtain early (E′) and late (A′) diastolic velocities. Isovolumic contraction time (IVCT) and isovolumic relaxation time (IVRT) were measured for both ventricles.

#### 2.3.2. Speckle Tracking Acquisition and Analysis

Myocardial deformation analysis was performed offline using the AutoStrain module of Philips QLab software (version 15.0; Philips Healthcare, Andover, MA, USA). Acquisition parameters were optimized to achieve high spatial and temporal resolution appropriate for neonatal STE. Only cardiac cycles with continuous and clearly visualized endocardial borders across all myocardial segments were considered acceptable. Standard apical views, including apical four-chamber (A4C), two-chamber (A2C), and three-chamber (A3C) projections, were acquired to allow comprehensive ventricular and atrial deformation assessment. Imaging depth was minimized and sector width restricted to improve tracking fidelity. Frame rates were maintained between 60 and 90 frames per second, in line with current neonatal STE recommendations. This frame rate range reflects a compromise between temporal and spatial resolution, which is particularly important in neonatal imaging. While higher frame rates improve temporal resolution, they may reduce spatial resolution and impair endocardial border delineation and tracking quality. Therefore, acquisition parameters were optimized individually to ensure reliable speckle-tracking analysis. For each view, at least three consecutive cardiac cycles were digitally stored for offline analysis.

Right ventricle deformation analysis was performed from an RV-focused A4C view. RV free-wall longitudinal strain (RV_FWSL) and RV four-chamber longitudinal strain (RV_4CSL) were obtained as established indices of RV systolic function (Figure 2A).

Left ventricle endocardial borders were automatically identified at end-diastole, with manual adjustments applied when necessary. A region of interest encompassing the full myocardial wall thickness was generated and optimized. Longitudinal strain analysis was performed using a 17-segment LV model as recommended by the ASE [8]. Global longitudinal strain (LV_GLS) was calculated as the average of peak negative strain values from all segments and expressed as a percentage of myocardial shortening (Figure 2B).

Atrial deformation analysis of the RA and LA was conducted using standard A4C views. Endocardial contours were manually traced at end-systole, followed by automated generation of a region of interest encompassing the atrial wall. Manual corrections were performed to exclude adjacent structures. The zero-strain reference point was set at end-diastole (onset of the QRS complex). For atrial deformation analysis, strain values were also derived using P-wave–based reference timing to allow a more physiologically accurate assessment of atrial contractile function, complementing the QRS-based reference used for reservoir function evaluation. Reservoir, conduit, and contractile strain components were derived for both atria, and global atrial strain values were calculated as the mean of all adequately tracked segments (Figure 2C,D).

#### 2.3.3. Intra-Observer Variability

Intra-observer variability for 2D and STE measurements was minimized through the use of a standardized acquisition and analysis protocol. All echocardiographic examinations and offline deformation analyses were performed by a single experienced investigator using the same ultrasound platform and software version throughout the study period. Image acquisition parameters, including frame rate, imaging depth, and sector width, were optimized in accordance with current recommendations, and only studies with optimal endocardial border definition were included. Measurements were performed following ASE guidelines, with consistent manual adjustments applied when required to ensure accurate contour tracking. This centralized approach was adopted to reduce measurement variability and enhance internal consistency, which is particularly important in neonates with EA, given the complex right heart geometry and elevated heart rates characteristic of this population.

### 2.4. Statistical Analysis

Statistical analysis was performed using SPSS software version 29.0 for Windows (IBM Corp., Armonk, NY, USA). Descriptive statistics were calculated for all variables and are presented as absolute and relative frequencies for categorical variables, and as mean ± standard deviation (range) for continuous variables. The distribution of continuous variables was assessed using the Kolmogorov–Smirnov test. As the majority of variables did not follow a normal distribution, non-parametric statistical methods were applied.

Comparisons between two groups were performed using the Mann–Whitney U test for continuous variables and the chi-square test for categorical variables. For comparisons involving more than two groups, the Kruskal–Wallis test was used. When statistically significant differences were identified, post hoc pairwise comparisons were conducted using Bonferroni-adjusted significance levels to control for multiple testing. The median test was additionally applied for selected variables where appropriate. All statistical tests were two-sided, and a *p*-value < 0.05 was considered statistically significant.

Receiver operating characteristic (ROC) curve analysis was performed to evaluate the diagnostic performance of echocardiographic strain parameters in discriminating neonates with Ebstein’s anomaly from healthy controls. ROC curves were constructed for parameters demonstrating statistically significant differences between groups. Diagnostic accuracy was quantified using the area under the curve (AUC), with corresponding 95% confidence intervals and *p*-values reported. The AUC was interpreted as follows: values between 0.7–0.8 indicated acceptable discrimination, 0.8–0.9 good discrimination, and >0.9 excellent diagnostic performance. Optimal cut-off values for each parameter were determined using Youden’s index (J = sensitivity + specificity − 1), which identifies the threshold that maximizes the combined sensitivity and specificity. Sensitivity and specificity values corresponding to the optimal cut-off were also calculated.

GraphPad Prism version 9.5.1. software (GraphPad Software, San Diego, CA, USA) was additionally used for graphical representation of selected results.

### 2.5. Objectives

The objectives of the study are as follows: (i) to compare conventional 2D echocardiographic parameters in controls and neonates with EA; (ii) to compare echocardiographic segmental and global strain parameters in controls and neonates with EA; and (iii) to evaluate the diagnostic performance of strain parameters.

## 3. Results

### 3.1. Description of Studied Groups

The study population consisted of 16 neonates diagnosed with EA and 26 healthy neonates serving as controls. The two groups were comparable in baseline perinatal characteristics, including sex distribution, gestational age, birth weight, and birth length, confirming appropriate cohort comparability. Despite similar perinatal profiles, neonates with EA demonstrated early indicators of compromised growth and cardiac function. Specifically, the EA group exhibited a significantly lower body surface area (BSA) z-score (*p* = 0.004), suggesting relative growth restriction (Table 1).

Marked hemodynamic impairment was evident: SaO_2_ was substantially reduced, and both mean BP and its z-score were significantly lower than in controls (*p* < 0.001) (Table 1). Heart rate (HR) tended to be higher, although not significantly, likely reflecting compensatory tachycardia. N-terminal pro-B-type natriuretic peptide (NT-proBNP) concentrations were more than doubled in the EA group (*p* < 0.001), indicating pronounced myocardial stress and early ventricular dysfunction. Additionally, the CT index, available only for the EA cohort, was elevated, consistent with cardiomegaly and right-sided dilation characteristic of the anomaly (Table 1).

Overall, these findings demonstrate that, despite similar perinatal characteristics, neonates with EA exhibit clear clinical, hemodynamic, and biochemical evidence of significant early cardiac compromise.

### 3.2. Characteristics of the Neonates from the Ebstein’s Anomaly Group

In the EA group (*n* = 16), no genetic syndromes or major comorbidities were identified. Fetal distress occurred in over one-third of cases (37.5%), and prenatal diagnosis was established in 43.8%. Blood gas analysis indicated mild acidemia (mean pH 7.33 ± 0.06) and elevated lactate levels (2.37 ± 1.82 mmol/L), accompanied by significant hypoxemia (mean pO_2_: 36.6mmHg; SO_2_: 82.9%), reflecting compromised perinatal oxygenation (Table 2).

Regarding anatomical severity, Carpentier type A morphology was most common (50%), and most neonates demonstrated moderate functional severity, with 68.8% classified as GOSE II. Associated structural defects were frequent, particularly atrial septal defects (87.6%). More than half of the infants exhibited arrhythmias, including sinus tachycardia, Wolff–Parkinson–White syndrome, and atrial tachycardia (each 18.8%) (Table 2).

Hemodynamic assessment revealed that RVFF was preserved in 56.3% of neonates, reduced in 18.8%, and absent in 25%. Importantly, 12.5% exhibited a devastating circular shunt, indicating severe hemodynamic compromise. A patent ductus arteriosus (PDA) was present in nearly all patients (93.7%) and closed spontaneously in the majority (81.3%) (Table 2).

Most neonates required intensive medical management, including high inspired oxygen concentrations (FiO_2_ 100%) (75%), milrinone (56.3%), diuretics (87.5%), sildenafil (81.3%), while inhaled nitric oxide (NO) was used in 6.3%. Prostaglandin E1 therapy was employed selectively (12.5%). Antiarrhythmic therapy (sotalol) was necessary in 25% of cases. Mean hospitalization duration was 12.5 ± 7.3 days, and neonatal mortality was 6.3%, with no surgical interventions required during the neonatal period (Table 2).

### 3.3. Conventional Right Ventricle and Left Ventricle Echocardiographic Characteristics of the Neonates from the Ebstein’s Anomaly Group at Birth

**Figure 3:**
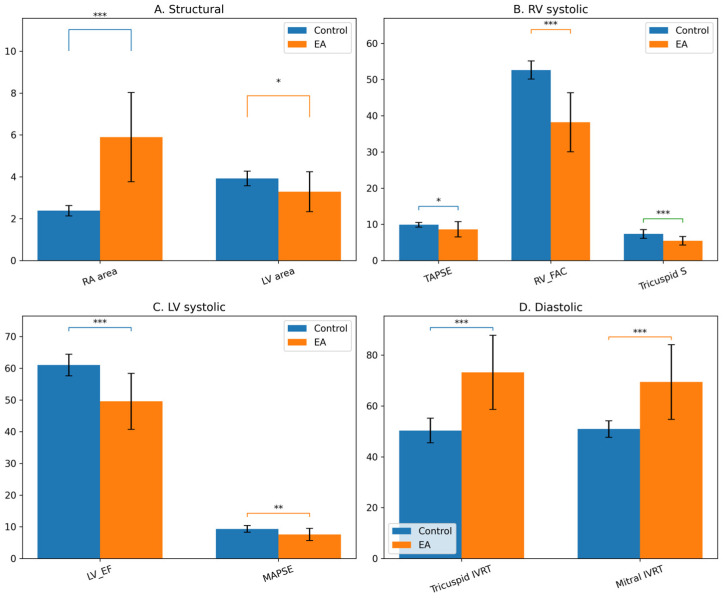
Comparison of structural and functional echocardiographic parameters between control neonates and those with Ebstein’s anomaly. Legend: (**A**) Structural remodeling (right atrial and left ventricular areas); (**B**) Right ventricular systolic function (TAPSE, RV_FAC, tricuspid S); (**C**) Left ventricular systolic function (LV_EF, MAPSE); (**D**) Diastolic function (tricuspid and mitral IVRT). Data are presented as mean ± SD. Asterisks indicate levels of statistical significance between groups (* *p* < 0.05, ** *p* < 0.01, *** *p *< 0.001).

Neonates with EA demonstrated profound structural and functional cardiac alterations compared with healthy controls (Table 3, Figure 3).

Structural remodeling was characterized by marked RA enlargement and relative compression of left-sided chambers. As illustrated in Figure 3A, RA area was significantly increased in the EA group, while LV area was reduced, reflecting ventricular interdependence and right-sided volume overload.

Right ventricular systolic function was significantly impaired. As shown in Figure 3B, TAPSE, RV_FAC, and tricuspid annular systolic velocity (S wave) were all significantly reduced in neonates with EA, indicating global RV systolic dysfunction. In addition to right-sided impairment, LV systolic function was also affected. Figure 3C demonstrates significantly lower LV_EF and MAPSE values in the EA group, supporting the presence of early secondary LV dysfunction. Diastolic function was similarly altered. As presented in Figure 3D, both RV IVRT and LV IVRT were significantly prolonged in neonates with EA, indicating impaired ventricular relaxation.

**Figure 4:**
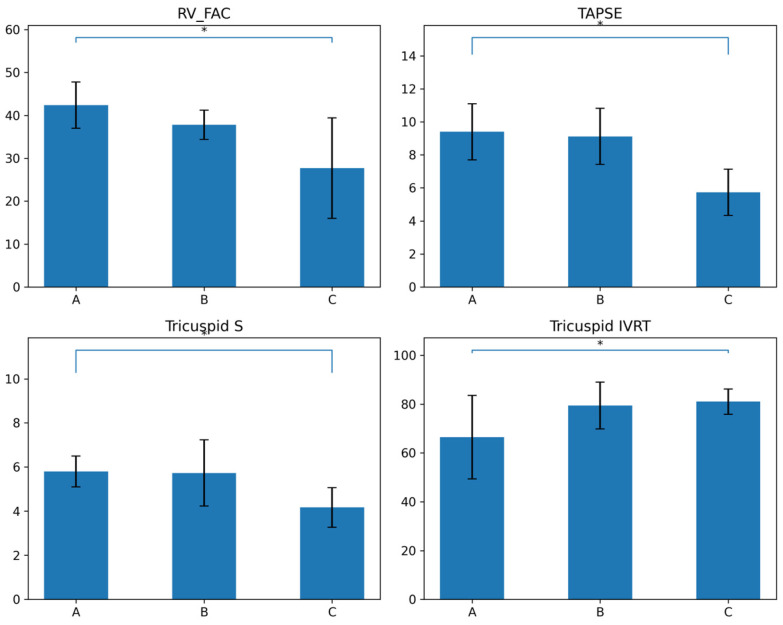
Comparison of echocardiographic functional parameters across Carpentier subtypes (A, B, C) in neonates with Ebstein’s anomaly. Bar graphs illustrate progressive deterioration in right ventricular systolic and diastolic function with increasing anatomical severity. * Data are presented as mean ± SD; error bars represent standard deviation. *p* < 0.05.

Across Carpentier subtypes, a progressive deterioration in RV function was observed (Supplementary Table S1, Figure 4). Detailed subgroup and segmental analyses are provided in Supplementary Tables S1 and S2.

As illustrated in Figure 4, RV_FAC showed a stepwise decline from Carpentier type A to type C, indicating worsening systolic performance with increasing anatomical severity. A similar trend was observed for TAPSE and tricuspid annular systolic velocity (S wave), both demonstrating reduced longitudinal RV function in more advanced subtypes. Diastolic dysfunction also progressed with disease severity. As shown in Figure 4, RV IVRT increased significantly from type A to type C, reflecting impaired RV relaxation. These findings highlight that functional echocardiographic parameters, particularly RV_FAC, TAPSE, tricuspid S, and RV IVRT, provide clear discrimination of disease severity across Carpentier subtypes and may be more sensitive indicators than structural measurements.

### 3.4. Echocardiographic Strain Indices in Control Group and Neonates with Ebstein’s Anomaly at Birth

Neonates with EA showed markedly impaired myocardial deformation compared with healthy controls. RV strain was significantly reduced across all segments (all *p* < 0.001), with the greatest impairment observed in the RV_FWSL and total RV_4CSL, indicating substantial early RV systolic dysfunction. LV strain was also significantly decreased in almost all segments, and LV_GLS was markedly reduced (LV_GLS −14.53% vs. −22.32% in controls, *p* < 0.001), demonstrating early involvement of LV systolic impairment despite the primarily right-sided defect (Table 4).

Atrial deformation was profoundly altered. Both RA and LA reservoir, conduit, and contractile strain values were significantly lower in the EA group (all *p* < 0.001), reflecting severe atrial dysfunction and reduced compliance (Table 4).

Overall, these findings demonstrate marked biventricular and biatrial deformation abnormalities in neonates with EA at birth, with the most pronounced impairment observed in right-sided chambers. The widespread reduction in strain underscores the significant hemodynamic burden and early myocardial involvement characteristic of this condition from the neonatal period.

Strain analysis demonstrated a progressive decline in RV and LV deformation with increasing Carpentier severity. RV strain indices were significantly reduced in all EA subgroups compared with controls (*p* < 0.001), with the most pronounced impairment in Carpentier C neonates across basal, mid, and apical RV segments, including RV_FWSL and RV_4CSL, reflecting severe loss of RV longitudinal shortening in advanced disease. LV segmental and global longitudinal strain also declined progressively from Carpentier A to Carpentier C, with the lowest values observed in Carpentier C neonates, indicating substantial secondary LV systolic dysfunction. The most pronounced reductions occurred in the basal inferoseptal, mid-lateral, inferior, and anterolateral segments. The stepwise decrease in LV_GLS across subtypes further supports the presence of biventricular impairment that parallels increasing anatomic severity. Atrial deformation was profoundly impaired across all EA subgroups. Right atrial strain parameters, reservoir, conduit, and contractile, were markedly reduced in all EA subgroups (all *p* < 0.001) and showed a clear stepwise deterioration toward type C, reflecting impaired atrial compliance and contractile function. Left atrial deformation demonstrated a similar, though less pronounced, pattern of deterioration. LA strain indices were significantly reduced across all EA subtypes, particularly for reservoir and conduit strain, with an additional decline in contractile strain evident in the most severe (Carpentier C) group (Supplementary Table S2).

Overall, these findings indicate that strain abnormalities worsen progressively from Carpentier A to C, with the most severe biventricular and biatrial dysfunction seen in Carpentier C neonates.

### 3.5. Diagnostic Performance of Strain Parameters

Receiver operating characteristic (ROC) analysis showed that deformation indices, especially LV_GLS and RA strain, offer excellent diagnostic discrimination, with potential applicability for early screening, risk stratification, and individualized management in neonatal EA.

**Figure 5:**
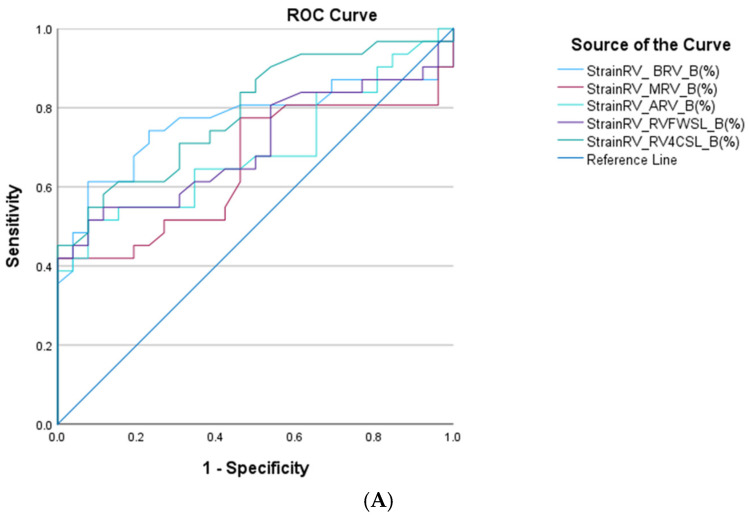

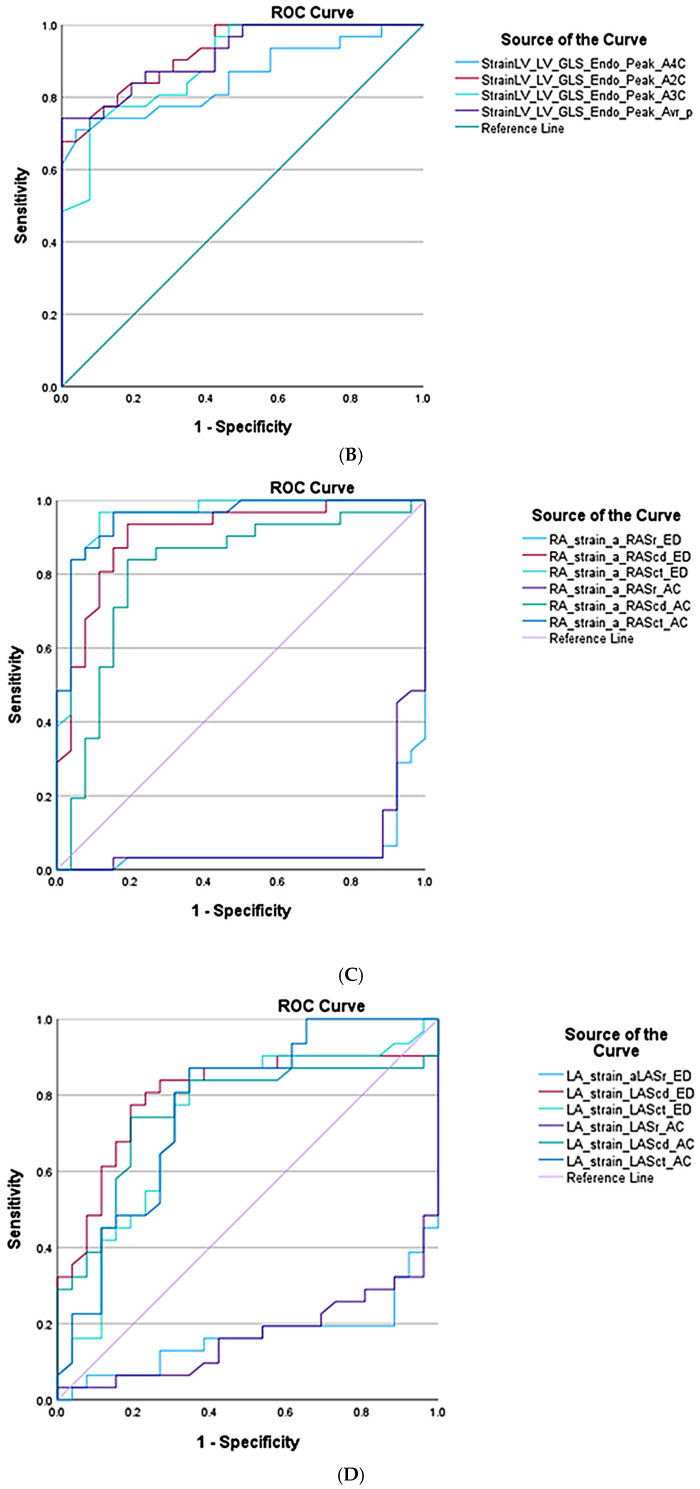
(**A**) Receiver operating characteristic (ROC) curve analysis of myocardial deformation parameters in neonates with Ebstein’s anomaly. ROC curves illustrating the diagnostic performance of speckle-tracking echocardiography–derived strain parameters in differentiating neonates with Ebstein’s anomaly from healthy controls. Right ventricular (RV) strain parameters, including RV_BRV_B: basal right ventricle segment longitudinal strain; RV_MRV_B: mid right ventricle segment longitudinal strain; RV_ARV_B: apical right ventricle segment longitudinal strain; RV free-wall longitudinal strain (RV_FWSL_B) and RV four-chamber longitudinal strain (RV_4CSL_B). The diagonal reference line represents the line of no discrimination (area under the curve = 0.5). Higher curves indicate better diagnostic performance of the corresponding parameter. (**B**) Receiver operating characteristic (ROC) curve analysis of myocardial deformation parameters in neonates with Ebstein’s anomaly. ROC curves illustrating the diagnostic performance of speckle-tracking echocardiography–derived strain parameters in differentiating neonates with Ebstein’s anomaly from healthy controls. Left ventricular (LV) strain parameters, including LV_GLS_A4C: left ventricular apical four-chamber longitudinal strain; LV_GLS_A2C: left ventricular apical two-chamber longitudinal strain; LV_GLS_A3C: left ventricular apical three-chamber longitudinal strain; LV_GLS_Avr: left ventricular global longitudinal strain. The diagonal reference line represents the line of no discrimination (area under the curve = 0.5). Higher curves indicate better diagnostic performance of the corresponding parameter. (**C**) Receiver operating characteristic (ROC) curve analysis of myocardial deformation parameters in neonates with Ebstein’s anomaly. ROC curves illustrating the diagnostic performance of speckle-tracking echocardiography–derived strain parameters in differentiating neonates with Ebstein’s anomaly from healthy controls. Right atrial (RA) strain parameters, including RASr_ED: right atrial reservoir strain by using the starting points of R-wave peak; RAScd_ED: right atrial conduit strain by using the starting points of R-wave peak; RASct_ED: right atrial contractile strain by using the starting points of R-wave peak; RASr_AC: right atrial reservoir strain by using the starting points of P-wave onset; RAScd_AC: right atrial conduit strain by using the starting points of P-wave onset; RASct_AC: right atrial contractile strain by using the starting points of P-wave onset. The diagonal reference line represents the line of no discrimination (area under the curve = 0.5). Higher curves indicate better diagnostic performance of the corresponding parameter. (**D**) Receiver operating characteristic (ROC) curve analysis of myocardial deformation parameters in neonates with Ebstein’s anomaly. ROC curves illustrating the diagnostic performance of speckle-tracking echocardiography–derived strain parameters in differentiating neonates with Ebstein’s anomaly from healthy controls. Left atrial (LA) strain parameters, including LASr_ED: left atrial reservoir strain by using the starting points of R-wave peak; LAScd_ED: left atrial conduit strain by using the starting points of R-wave peak; LASct_ED: left atrial contractile strain by using the starting points of R-wave peak; LASr_AC: left atrial reservoir strain by using the starting points of P-wave onset; LAScd_AC: left atrial conduit strain by using the starting points of P-wave onset; LASct_AC: left atrial contractile strain by using the starting points of P-wave onset. The diagonal reference line represents the line of no discrimination (area under the curve = 0.5). Higher curves indicate better diagnostic performance of the corresponding parameter.

Right ventricular longitudinal strain parameters demonstrated good diagnostic performance, with area-under-the-curve (AUC) values ranging from 0.653 to 0.790. Among these indices, RV_4CSL emerged as the most accurate RV marker, achieving an AUC of 0.790 with an optimal cut-off value of −19.2%. These findings underscore the sensitivity of RV myocardial deformation to early right-sided functional impairment in neonatal EA (Figure 5A), (Table 5).

Left ventricular longitudinal strain indices exhibited excellent diagnostic accuracy, with AUC values ranging from 0.853 to 0.924. Among the individual views, LV_GLS_A2C provided the strongest discriminatory performance (AUC 0.924; optimal cut-off −20.25%). The LV_GLS also demonstrated robust diagnostic accuracy (AUC 0.919; cut-off −21.05%). Collectively, these findings highlight the high sensitivity of LV myocardial deformation to early pathological changes, despite EA being predominantly a right-sided cardiac defect (Figure 5B), (Table 5).

Right atrial deformation indices showed excellent diagnostic performance, with RA conduit strain (RA_Scd_ED) achieving an AUC of 0.907 (cut-off −12.15%) and RA contractile strain (RA_Sct_ED) demonstrating the highest overall discriminatory accuracy (AUC 0.958; cut-off −13.9%). These results highlight severe RA mechanical dysfunction as a key feature of neonatal EA (Figure 5C), (Table 5).

Left atrial deformation parameters demonstrated moderate to good diagnostic performance, with conduit strain (LA_Scd_ED) achieving an AUC of 0.802 (cut-off −15.15%) and contractile strain (LA_Sct_ED) yielding an AUC of 0.736 (cut-off −24.50%). Although less discriminatory than RA strain indices, LA deformation parameters effectively differentiated neonates with EA from controls and provide complementary insight into left-sided atrial involvement (Figure 5D), (Table 5).

## 4. Discussion

The present study demonstrates that STE enables early detection of extensive biventricular and biatrial myocardial dysfunction in neonates with EA, including abnormalities not fully captured by conventional echocardiographic assessment, thereby providing incremental diagnostic value.

### 4.1. Early Systemic and Myocardial Compromise in Neonatal EA

A principal finding of the present study is the presence of marked systemic and myocardial compromise in neonates with EA despite preserved gestational age and birth weight. Profound hypoxemia, reduced systemic arterial pressures, and markedly elevated serum NT-proBNP levels collectively indicate severe circulatory derangement in the early postnatal period. These abnormalities are mechanistically attributable to the combined effects of severe TR, pronounced RA dilatation, and ineffective RV forward output, resulting in impaired pulmonary blood flow and compromised systemic perfusion. Such pathophysiological features have been consistently reported in neonatal cohorts and are strongly associated with early postnatal morbidity and mortality, particularly during the transitional circulation when PVR remains elevated and RV functional reserve is limited [1,2,9,10,11,12,13,14,15,16,17]. The marked elevation of NT-proBNP observed in our cohort further underscores the presence of intrinsic myocardial stress and early neurohormonal activation. Although formal correlation analyses between NT-proBNP levels and strain parameters were not performed due to the limited sample size, the markedly elevated NT-proBNP levels observed in the EA group, together with significantly reduced deformation indices, support the presence of early myocardial dysfunction. This parallel alteration suggests that myocardial deformation parameters may reflect the degree of myocardial stress, although larger studies are required to confirm this relationship. Accumulating histopathological and clinical evidence indicates that EA is associated with primary myopathic alterations of the RV myocardium, including reduced myocardial fiber density, fiber disarray, and interstitial fibrosis, affecting both the atrialized and FRV components. These observations support the conceptualization of EA as a combined valvular-myocardial disorder rather than an isolated TV malformation [1,2,11,15,18,19]. During the immediate postnatal period, persistently elevated PVR further exacerbates RV dysfunction by limiting antegrade pulmonary flow, frequently resulting in ductal-dependent pulmonary circulation in severe neonatal phenotypes [1,11,16].

### 4.2. Anatomical Severity Versus Functional Hemodynamics

Large cohort and multicenter studies consistently demonstrate that anatomical classification alone is insufficient to reliably predict neonatal outcomes unless accompanied by markers of impaired RV performance or absent RVFF [1,11,19].

Although the majority of neonates in the present cohort exhibited Carpentier type A or B morphology and only moderate echocardiographic severity as assessed by the GOSE score, clinically relevant circulatory instability was frequently observed. Notably, a subset of patients demonstrated absent RVFF and circular shunt physiology, highlighting a substantial dissociation between anatomical descriptors and functional hemodynamic severity in neonatal EA. This discordance was first systematically described by Celermajer and colleagues, who demonstrated that neonatal outcomes were more closely associated with cardiomegaly, ventricular interdependence, and FRV performance than with anatomical classification alone [14,15]. Subsequent multicenter and contemporary cohort studies have consistently corroborated these findings, indicating that anatomical severity in isolation does not reliably predict neonatal outcomes unless accompanied by markers of impaired RV function, absent RVFF, or adverse postnatal circulatory physiology [1,11,17,19]. Collectively, these observations reinforce the concept that functional hemodynamic status during early postnatal adaptation represents a more critical determinant of clinical trajectory than anatomical severity per se. They also underscore the inherent limitations of anatomy-based classification systems when applied in isolation in the neonatal period.

### 4.3. Impact of Associated Shunts and Transitional Physiology

The frequent coexistence of atrial septal defects and PDA in the present cohort reflects the broader spectrum of congenital cardiac abnormalities associated with EA and underscores their physiological relevance during the neonatal period. Right-to-left interatrial shunting may transiently preserve systemic cardiac output in the setting of impaired RVFF; however, this compensatory mechanism concurrently exacerbates systemic arterial desaturation and contributes to RA volume overload. This RA volume overload, largely driven by interatrial communication, may significantly influence atrial remodeling and deformation parameters. These findings are consistent with previous studies highlighting the interplay between structural defects and atrial mechanics in congenital heart disease [20]. Similarly, ductal patency may be essential for maintaining pulmonary blood flow in neonates with severely compromised RV output, yet its net hemodynamic impact is highly context dependent, being influenced by PVR, the direction and magnitude of ductal shunting, and intrinsic RV functional capacity [1,11,16,21]. The heterogeneous requirement for prostaglandin E1 therapy observed in our cohort reflects this delicate physiological balance and is consistent with previously reported neonatal management strategies in EA [1,21]. Arrhythmias were identified in a substantial proportion of neonates in the present cohort, including Wolff–Parkinson–White syndrome and supraventricular tachyarrhythmias, in keeping with the well-established association between EA and electrical conduction abnormalities. Accessory atrioventricular pathways and atrial tachyarrhythmias have been reported across all age groups in this condition, reflecting abnormalities of atrioventricular junctional development and marked RA enlargement. In the neonatal period, this arrhythmic burden may further compromise cardiac output by disrupting atrioventricular synchrony and limiting effective ventricular filling, thereby contributing to hemodynamic instability during a critical phase of postnatal circulatory adaptation [2,6,13,21,22].

### 4.4. Biventricular Dysfunction and Ventricular Interdependence

Neonates with EA demonstrated pronounced right-sided structural remodeling at birth, characterized by severe RA enlargement and marked reduction of the FRV cavity, accompanied by significantly smaller LA and LV dimensions. These findings are consistent with the established pathophysiological paradigm of EA, whereby RV atrialization and severe TR lead to progressive right-sided dilatation, enhanced ventricular interdependence, and extrinsic compression of left-sided cardiac chambers [1,11,14].

Conventional echocardiographic assessment revealed significant RV systolic and diastolic dysfunction, as evidenced by reduced TAPSE, diminished RV_FAC, depressed tricuspid annular tissue Doppler velocities, and prolongation of isovolumic time intervals. However, substantial interindividual variability was observed, reflecting the inherent limitations of conventional RV functional indices in this population. These parameters are highly load dependent and are further confounded by abnormal RV geometry, the presence of atrialized myocardium, and altered interventricular interactions. In neonates, such limitations are compounded by elevated heart rates, small chamber dimensions, and rapidly evolving loading conditions during early postnatal adaptation, thereby reducing the reliability of conventional indices for comprehensive RV functional assessment [4,8].

Importantly, LV systolic and diastolic dysfunction was also evident, as indicated by reduced LV_EF, diminished MAPSE, and impaired diastolic filling indices. These abnormalities most likely reflect the effects of ventricular interdependence, interventricular septal displacement, and extrinsic LV compression by markedly dilated right-sided chambers, rather than primary LV myocardial disease [11,19,21]. Increasing evidence from fetal and neonatal studies suggests that LV dysfunction is common in EA, frequently underestimated by conventional echocardiography, and represents a clinically relevant contributor to global circulatory compromise and adverse early outcomes, particularly when diagnostic attention is focused predominantly on RV pathology [11,19,23]. Extending these observations, the present findings demonstrate that biventricular dysfunction is already present at birth, even among neonates with predominantly moderate anatomical severity, underscoring the importance of comprehensive biventricular functional assessment during the early neonatal period.

### 4.5. Functional Severity Across Carpentier Subtypes

When stratified according to Carpentier classification, a stepwise worsening of right-sided structural remodeling and functional impairment was observed from type A to type C. While structural dimensions alone did not consistently discriminate between morphological subtypes, functional indices revealed more distinct and clinically meaningful differences. Neonates with Carpentier type C morphology demonstrated the most severe RV systolic dysfunction, characterized by markedly reduced RV_FAC, diminished TAPSE, depressed tricuspid annular systolic velocities, and significantly prolonged RV relaxation intervals.

Importantly, LV systolic and diastolic function deteriorated in parallel with increasing anatomical severity. Neonates with Carpentier type C morphology exhibited the lowest LV_EF and the most prolonged LV isovolumic time intervals, indicating impaired biventricular performance. Collectively, these findings underscore the limited prognostic utility of anatomical classification when applied in isolation and are consistent with prior multicenter studies demonstrating that neonatal outcomes in EA are more closely related to FRV performance and the presence of antegrade pulmonary blood flow than to morphological severity alone [1,11,19].

### 4.6. Incremental Value of Myocardial Deformation Imaging

A major strength of the present study is the comprehensive evaluation of myocardial deformation using STE. Deformation imaging has shown incremental diagnostic and prognostic value in congenital heart disease [24,25]. Neonates with EA exhibited markedly reduced RV longitudinal strain across all myocardial segments, with the most pronounced impairment observed in RV_FWSL and RV_4CSL, indicating substantial early loss of longitudinal RV contractile function. These findings are consistent with prior pediatric and adult studies demonstrating that RV strain represents a sensitive marker of disease severity and functional impairment in EA and frequently provides incremental information beyond conventional echocardiographic indices [5,6].

Left ventricle myocardial deformation was also significantly impaired, with markedly reduced LV_GLS and diffuse segmental strain abnormalities, particularly in septal and lateral segments. These findings support secondary LV systolic dysfunction mediated by ventricular interdependence and abnormal septal mechanics and are consistent with reports in pediatric and adult EA linking reduced LV strain to worse functional status and outcomes. Thus, LV involvement in neonatal EA is clinically relevant and detectable at birth by deformation imaging. The reduction in LV_GLS likely reflects both mechanical and intrinsic mechanisms. Right-sided dilation and interventricular septal displacement can compress the LV and impair filling, producing the characteristic “pancaked” configuration. Intrinsic myocardial abnormalities, including altered fiber architecture and fibrosis, may also contribute [5,6,23].

Atrial deformation analysis revealed profound RA dysfunction, with severe impairment of reservoir, conduit, and contractile strain across all EA subgroups. RA strain worsened with increasing Carpentier severity, indicating impaired compliance and reduced contractile reserve, whereas LA strain was also reduced, likely reflecting downstream effects of ventricular dysfunction and altered filling. Because atrial impairment may reduce ventricular preload and increase arrhythmic vulnerability, atrial strain appears to be a clinically meaningful marker of disease severity and prognosis in EA. The choice of zero-strain reference may influence atrial strain values and their phasic interpretation; QRS-based reference emphasizes reservoir function, whereas P-wave–based reference more closely reflects contractile function [6,7]. Consistent with prior reports, atrial strain has emerged as a clinically meaningful marker of disease severity and prognosis in congenital heart disease, including EA, particularly with respect to arrhythmic burden and heart failure progression [26].

### 4.7. Strain Patterns Across Carpentier Severity

Strain parameters showed a stepwise deterioration from Carpentier type A to C, with type C neonates demonstrating the greatest impairment in RV_FWSL, RV_4CSL, LV_GLS, and atrial deformation indices. These findings suggest that deformation imaging reflects physiological severity more closely than structural measurements alone and may improve early functional stratification beyond anatomy-based classification systems.

### 4.8. Diagnostic Performance and Clinical Utility of Strain Parameters

ROC analysis demonstrated strong diagnostic performance of myocardial deformation parameters, with LV_GLS and RA strain showing superior discriminatory ability compared with conventional echocardiographic measures. RV_4CSL was the most robust RV deformation marker, while RA conduit and contractile strain achieved the highest overall diagnostic accuracy, highlighting the central role of atrial mechanical dysfunction in the neonatal EA phenotype. Collectively, these findings indicate that myocardial deformation imaging, especially LV_GLS and RA strain, adds substantial value for early diagnosis, functional characterization, and risk stratification in neonatal EA.

### 4.9. Clinical Implications

These findings highlight the incremental value of myocardial deformation imaging for the early detection of biventricular and biatrial dysfunction, supporting its role in comprehensive functional assessment and early risk stratification in neonatal EA. STE provides sensitive, largely geometry-independent markers of myocardial performance that may improve individualized management and support the timing of referral and intervention. Deformation parameters may also serve as useful markers for longitudinal follow-up and prognostic assessment.

### 4.10. Limitations

This study has several limitations. Its single-center, retrospective design and relatively small sample size may limit generalizability and introduce selection bias, as only neonates with complete, high-quality echocardiographic datasets were included. The study was not prospectively powered for subgroup analyses across Carpentier classes; therefore, these findings should be interpreted as exploratory and hypothesis-generating. Inter-observer variability was not assessed because all acquisitions and offline analyses were performed by a single experienced investigator; however, a standardized protocol, adherence to ASE recommendations, and strict image quality criteria were applied to minimize measurement variability. In addition, frame rates near the lower end of the acquisition range may have introduced partial temporal undersampling in neonates with high heart rates, and atrial strain values may vary depending on the selected zero-strain reference. Nevertheless, the consistency of findings across clinical, biochemical, conventional echocardiographic, and myocardial deformation parameters supports the internal coherence of the results.

### 4.11. Future Directions

Future multicenter studies with larger cohorts and longitudinal follow-up are needed to define clinically meaningful strain thresholds, assess reproducibility, and validate prognostic value in neonatal EA. Such work may support integration of myocardial deformation imaging into standardized diagnostic and risk stratification frameworks.

## 5. Conclusions

Speckle-tracking echocardiography provides a sensitive and clinically relevant tool for the early detection of myocardial dysfunction in neonatal EA, offering incremental value beyond conventional echocardiographic assessment. This study demonstrates that myocardial deformation imaging captures the complex functional phenotype of neonatal EA at birth, including RV dysfunction, early LV involvement, and significant atrial impairment. These findings support a more comprehensive, function-oriented approach to early evaluation in this high-risk population. Importantly, this work should be interpreted as a functional characterization study that establishes a reference framework for myocardial deformation patterns in neonatal EA. In the absence of established neonatal reference values, these results contribute to a better understanding of early myocardial mechanics and may serve as a foundation for future risk stratification strategies. Further multicenter studies with larger cohorts and longitudinal follow-up are required to validate these findings, define clinically relevant strain thresholds, and determine the prognostic value of deformation parameters in neonatal EA.

## Figures and Tables

**Table 1:** General characteristics of neonates in study groups.

Variable	Control Group (*n* = 26)	Ebstein’s Anomaly Group (*n* = 16)	*p*-Value
Sex (male%)	58%	63%	0.758 *
Gestational age (weeks)	38.85 ± 2.34	38.81 ± 1.91	0.957 **
BW (g)	3484 ± 246	3260 ± 451	0.126 **
BL (cm)	54.46 ± 2.08	53.13 ± 3.22	0.114 **
BSA (m^2^)	0.232 ± 0.011	0.220 ± 0.021	0.070 **
BSA z-score	−0.288 ± 0.214	−0.682 ± 0.428	0.004 **
SaO_2_ (%)	-	84.9 ± 14.1	-
HR (bpm)	135.8 ± 5.0	147.3 ± 28.8	0.199
HR z-score	0.541 ± 0.376	1.346 ± 2.019	0.198
mBP (mmHg)	61.65 ± 3.59	48.63 ± 8.02	<0.001
mBP z-score	0.230 ± 0.428	−1.318 ± 0.882	<0.001
NT-proBNP (pg/mL)	731.3 ± 215.4	1933.1 ± 993.4	<0.001
CT index	-	0.6456 ± 0.0868	-

(* Chi square bivariate test, ** Independent-Samples Mann–Whitney U Test). BW: birth weight; BL: birth length; BSA: body surface area; BSA z-score: body surface area z score; SaO_2_: peripheral oxygen saturation; HR: heart rate; HR z-score: heart rate z score; mBP: median blood pressure; mBP z-score: mean blood pressure z score; NT-proBNP: N-terminal pro-B-type natriuretic peptide; CT index: cardio-thoracic index; *n*: number.

**Table 2:** Characteristics of the neonates from the Ebstein’s anomaly group.

Variable	Results (Ebstein’s Anomaly Group, *n* = 16)
Genetic syndrome	0% (none)
Comorbidities	0% (none)
Fetal distress	37.5% yes (6/16)
Prenatal diagnosis	43.8% yes (7/16)
pH (Astrup)	7.33 ± 0.06 (7.18–7.43)
Lactic acid (mmol/L) (Astrup)	2.37 ± 1.82 (0.7–6.7)
pO_2_ (mmHg) (Astrup)	36.6 ± 12.1 (16–58)
pCO_2_ (mmHg) (Astrup)	44.8 ± 10.5 (32–70.7)
BE (Astrup)	−0.6 ± 2.86 (−6.4–2.0)
SO_2_ (%) (Astrup)	82.9 ± 17.2 (40–98)
Carpentier type	A: 50% (*n* = 8) B: 31.3% (*n* = 5) C: 18.8% (*n* = 3)
GOSE index	0.71 ± 0.25 (0.40–1.40)
GOSE	I: 25% (*n* = 4) II: 68.8% (*n* = 11) III: 6.3% (*n* = 1)
Structural associated lesions	• Small ASD 68.8% • Moderate/Large ASD 18.8% • VSD 6.3% • Mitral regurgitation ≥ mild: 12.6% (1 mild, 1 moderate)
Electrical anomalies	• Sinusal tachycardia 18.8% • WPW 18.8% • Atrial tachycardia 18.8% • SVT 6.3% • AVRT 6.3% • AVB 0%
Hemodynamics in neonatal period	• RVFF at birth ○ absent 25% ○ reduced 18.8% ○ present-efficient 56.3% • Circular shunt ○ no 68.8% ○ tolerated 18.8% ○ devastating 12.5% • PDA at birth ○ small 68.8% ○ large 25% ○ absent 6.3% • PDA closure: ○ spontaneously 81.3% ○ pharmacological 6.3% ○ after-PGE1 cessation 6.3%
Therapy after birth	• PGE1: 12.5% • FiO_2_ 100%: 75% • O_2_ supplementation: 56.3% • NO: 6.3% • Sildenafil: 81.3% • Milrinone: 56.3% • Diuretics: 87.5% • Antiarrhythmics (Sotalol): 25% • Aspenter: 12.5%
Hospitalization days	12.5 ± 7.3 (1–26)
Neonatal death	6.3% (1/16)
Surgical correction (neonatal)	0%

pH: acidity or alkalinity of blood; pO_2_: partial pressure of oxygen; pCO_2_: partial pressure of carbon dioxide; BE: base excess; SO_2_: oxygen saturation; Carpentier classification of Ebstein’s anomaly: type A, type B, type C; GOSE index: Great Ormond Street Echocardiography Score; GOSE: Great Ormond Street Echocardiography Score grade; ASD: atrial septal defect; VSD: ventricular septal defect; WPW: Wolff–Parkinson–White syndrome; SVT: supraventricular tachycardia; AVRT: atrioventricular re-entry tachycardia; AVB: atrioventricular block; RVFF: right ventricular forward flow; PDA: patent ductus arteriosus; PGE1: prostaglandin E 1; FiO_2_: percentage of oxygen participating in gas-exchange; O_2_: oxygen; NO: nitric oxide; *n*: number; %: percentage.

**Table 3:** Structural and functional echocardiographic parameters in controls and Ebstein’s anomaly neonates at birth.

Variable	Control Group (*n* = 26)	EA Group (*n* = 16)	*p*-Value
Structural echocardiographic parameters			
RA area (cm^2^)	2.38 ± 0.25 (2.0–3.0)	5.90 ± 2.13 (2.6–9.4)	<0.001 **
RV/FRV area (cm^2^)	3.49 ± 0.53 (2.8–4.8)	2.84 ± 1.29 (0.9–6.1)	0.036 *
LA area (cm^2^)	2.77 ± 0.26 (2.3–3.5)	2.24 ± 0.69 (1.2–3.8)	0.002 **
LV area (cm^2^)	3.92 ± 0.35 (3.3–4.5)	3.29 ± 0.95 (1.6–5.1)	0.022 *
RV index	15.10 ± 2.18 (11.7–19.2)	12.94 ± 5.34 (3.7–25.4)	0.083
LV index	16.90 ± 1.64 (14.0–19.5)	15.02 ± 3.96 (7.0–21.3)	0.070
RA index	10.25 ± 0.96 (9.1–13.1)	27.10 ± 10.41 (12.8–52.2)	<0.001 **
GOSE index	—	0.71 ± 0.25 (0.4–1.4)	—
TV annulus (mm)	—	14.81 ± 2.12 (11.4–19.0)	—
TV annulus z-score	—	2.06 ± 1.26 (−0.01–4.17)	—
PV annulus (mm)	—	6.97 ± 1.89 (0.7–9.4)	—
PV annulus z-score	—	−0.87 ± 0.79 (−1.9–1.5)	—
Functional echocardiographic parameters			
TAPSE (mm)	9.89 ± 0.64 (8.8–11.2)	8.62 ± 2.11 (4.5–12.4)	0.018 *
RV_FAC (%)	52.63 ± 2.50 (49.5–59.5)	38.22 ± 8.17 (15–48.7)	<0.001 **
Tricuspid_E/A	1.03 ± 0.18 (0.75–1.45)	0.83 ± 0.13 (0.6–1.1)	<0.001 **
Tricuspid_E′ (cm/s)	13.65 ± 2.46 (9.8–18.1)	9.95 ± 3.06 (4.7–16.2)	<0.001 **
Tricuspid_A′ (cm/s)	12.61 ± 2.49 (8.8–18.7)	10.29 ± 2.43 (5.9–13.7)	0.011 *
Tricuspid_S (cm/s)	7.35 ± 1.21 (5.2–10.1)	5.47 ± 1.17 (3.2–8.4)	<0.001 **
Tricuspid_E/E′	0.05 ± 0.01 (0.04–0.07)	0.06 ± 0.02 (0.04–0.10)	0.257
Tricuspid_IVCT (ms)	50.27 ± 4.84 (44–60)	64.38 ± 14.93 (34–88)	<0.001 **
Tricuspid_IVRT (ms)	50.35 ± 4.84 (45–62)	73.25 ± 14.59 (45–100)	<0.001 **
LV_EF (%)	61.02 ± 3.41 (56–70)	49.58 ± 8.83 (25–60)	<0.001 **
MAPSE (mm)	9.33 ± 1.06 (7.5–11.2)	7.60 ± 1.92 (3.4–10.5)	0.003 **
Mitral_E/A	1.07 ± 0.17 (0.6–1.3)	0.92 ± 0.19 (0.6–1.3)	0.011 *
Mitral_E′ (cm/s)	14.48 ± 3.03 (8.1–19.4)	11.66 ± 4.06 (6.2–19.6)	0.018 *
Mitral_A′ (cm/s)	12.61 ± 1.80 (9.6–15.7)	10.70 ± 2.78 (6.6–14.9)	0.014 *
Mitral_S (cm/s)	7.03 ± 0.68 (5.9–8.2)	5.42 ± 1.14 (3.0–8.6)	<0.001 **
Mitral_E/E′	0.05 ± 0.01 (0.04–0.10)	0.05 ± 0.01 (0.03–0.09)	0.205
Mitral_IVCT (ms)	50.42 ± 3.30 (45–55)	65.25 ± 15.56 (37–90)	<0.001 **
Mitral_IVRT (ms)	50.92 ± 3.22 (46–56)	69.44 ± 14.72 (40–90)	<0.001 **

Data are expressed as mean ± SD (range). Significance tested by Mann–Whitney U test; *p* < 0.05 considered significant. * Independent-Samples Kruskal–Wallis Test; ** Independent-Samples Median Test. EA: Ebstein’s anomaly; RA: right atrium; RV: right ventricle; FRV: functional right ventricle; LA: left atrium; LV: left ventricle; GOSE: Great Ormond Street Echocardiography Score; TV: tricuspid valve; PV: pulmonary valve; TAPSE: tricuspid annular plain systolic excursion; RV_FAC: right ventricle fractional change area; tricuspid_E/A; the ratio between early tricuspid inflow velocity and late tricuspid inflow velocity; tricuspid_E’: tricuspid annular early diastolic velocity (E’wave) obtained by tissue Doppler echocardiography; tricuspid_A’: tricuspid annular late diastolic velocity (A’wave) obtained by tissue Doppler echocardiography; tricuspid_S: tricuspid annular systolic velocity (S wave) obtained by tissue Doppler echocardiography: tricuspid E/E’: the ratio between early tricuspid inflow velocity and tricuspid annular early diastolic velocity; tricuspid_IVCT: right ventricle isovolumic contraction time; tricuspid_IVRT: right ventricle isovolumic relaxation time; LV_EF: left ventricle ejection fraction; MAPSE: mitral annular plain systolic excursion, mitral_E/A: the ratio between early mitral inflow velocity and late mitral inflow velocity; mitral_E’: mitral annular early diastolic velocity (E’wave) obtained by tissue Doppler echocardiography; mitral_A’: mitral annular late diastolic velocity (A’wave) obtained by tissue Doppler echocardiography; mitral_S: mitral annular systolic velocity (S wave) obtained by tissue Doppler echocardiography; mitral_E/E’: the ratio between early mitral inflow velocity and mitral annular early diastolic velocity (E/E’); mitral_IVCT: left ventricle isovolumic contraction time; mitral_IVRT: left ventricle isovolumic relaxation time; *n*: number.

**Table 4:** Comprehensive strain echocardiographic indices in controls and Ebstein’s anomaly neonates at birth.

Variable	Control Group (*n* = 26) Mean ± SD	EA Group (*n* = 16) Mean ± SD	*p*-Value
Right ventricle segmental strain			
RV_BRV (%)	−30.6 ± 5.9	−17.5 ± 8.3	<0.001
RV_MRV (%)	−24.8 ± 4.7	−16.3 ± 7.8	<0.001
RV_ARV (%)	−24.63 ± 4.1	−17.1 ± 7.3	<0.001
RV_FWSL (%)	−26.6 ± 4.3	−17.3 ± 7.8	<0.001
RV_4CSL (%)	−23.9 ± 2.9	−15.6 ± 5.5	<0.001
Left Ventricle segmental strain			
LV_BIS (%)	−18.16 ± 3.95	−10.04 ± 5.20	<0.001
LV_MIS (%)	−21.56 ± 4.38	−14.94 ± 5.01	<0.001
LV_AIS (%)	−25.13 ± 4.75	−20.58 ± 5.94	0.020
LV_BAL (%)	−26.83 ± 7.14	−15.39 ± 8.62	<0.001
LV_MAL (%)	−18.65 ± 2.87	−11.02 ± 4.62	<0.001
LV_AAL (%)	−22.73 ± 5.29	−15.39 ± 8.82	0.030
LV_BI (%)	−20.09 ± 4.14	−12.88 ± 6.48	<0.001
LV_MI (%)	−20.61 ± 4.49	−13.48 ± 3.65	<0.001
LV_AI (%)	−24.57 ± 3.88	−16.62 ± 7.11	<0.001
LV_BA (%)	−21.78 ± 4.39	−13.53 ± 5.46	<0.001
LV_MA (%)	−21.19 ± 3.49	−12.08 ± 4.47	<0.001
LV_AA (%)	−22.94 ± 9.86	−16.28 ± 6.05	0.023
LV_BIL (%)	−23.37 ± 6.09	−15.76 ± 9.49	<0.001
LV_MIL (%)	−20.79 ± 5.83	−10.91 ± 4.46	<0.001
LV_AL (%)	−28.73 ± 30.28	−16.54 ± 4.84	<0.001
LV_BAS (%)	−20.23 ± 3.45	−11.63 ± 5.85	0.001
LV_MAS (%)	−21.02 ± 3.87	−14.19 ± 4.81	<0.001
LV_AA^2^ (%)	−22.12 ± 5.02	−21.80 ± 8.21	0.551
LV_GLS_A4C (%)	−22.24 ± 2.29	−14.33 ± 3.91	<0.001
LV_GLS_A2C (%)	−22.13 ± 2.09	−14.19 ± 3.93	<0.001
LV_GLS_A3C (%)	−21.84 ± 2.50	−15.08 ± 3.87	<0.001
LV_GLS (%)	−22.32 ± 1.94	−14.53 ± 3.59	<0.001
Right atrium strain analysis			
RA_Sr_ED (%)	36.93 ± 8.88	12.49 ± 5.16	<0.001
RA_Scd_ED (%)	−20.06 ± 5.52	−7.34 ± 3.45	<0.001
RA_Sct_ED (%)	−19.20 ± 6.11	−4.90 ± 2.82	<0.001
RA_Sr_AC (%)	34.12 ± 8.91	12.47 ± 5.16	<0.001
RA_Scd_AC (%)	−16.47 ± 8.96	−7.67 ± 5.85	<0.001
RA_Sct_AC (%)	−17.40 ± 5.15	−3.23 ± 3.82	<0.001
Left atrium strain analysis			
LA_Sr_ED (%)	37.49 ± 8.23	19.08 ± 12.95	<0.001
LA_Scd_ED (%)	−23.04 ± 4.32	−12.72 ± 5.29	<0.001
LA_Sct_ED (%)	−17.23 ± 7.55	−9.36 ± 5.58	0.002
LA_Sr_AC (%)	34.27 ± 6.93	20.03 ± 6.09	<0.001
LA_Scd_AC (%)	−21.13 ± 4.37	−12.94 ± 5.52	<0.001
LA_Sct_AC (%)	−15.58 ± 6.48	−7.71 ± 5.01	<0.001

Negative strain values indicate myocardial shortening (more negative = better deformation). Data are expressed as mean ± SD (range). Significance tested by Mann–Whitney U test; *p* < 0.05 considered significant. EA: Ebstein’s anomaly; RV_BRV: basal right ventricle segment; RV_MRV: mid right ventricle segment; RV_ARV: apical right ventricle segment; RV_FWSL: right ventricle free wall strain; RV_4CSL: right ventricle apical four-chamber view total strain; LV_BIS: left ventricle basal inferoseptal segment; LV_MIS: left ventricle mid inferoseptal segment; LV_AIS: left ventricle apical inferoseptal segment; LV_BAL: left ventricle basal anterolateral segment; LV_MAL: left ventricle mid anterolateral segment; LV_AAL: left ventricle apical anterolateral segment; LV_BI: left ventricle basal inferior segment; LV_MI: left ventricle mid inferior segment; LV_AI: left ventricle apical inferior segment; LV_BA: left ventricle basal anterior segment; LV_MA: left ventricle mid anterior segment; LV_AA: left ventricle apical anterior segment (assessed from apical two-chamber view); LV_BIL: left ventricle basal inferolateral segment; LV_MIL: left ventricle mid inferolateral segment; LV_AL: left ventricle apical lateral segment; LV_BAS: left ventricle basal anteroseptal segment; LV_MAS: left ventricle mid anteroseptal segment; LV_AA2: left ventricle apical anterior segment (assessed from apical three-chamber view); LV_GLS_A4C: left ventricular apical four-chamber longitudinal strain; LV_GLS_A2C: left ventricular apical two-chamber longitudinal strain; LV_GLS_A3C: left ventricular apical three-chamber longitudinal strain; LV_GLS: left ventricular global longitudinal strain; RA_Sr_ED: right atrial reservoir strain by using the starting points of R-wave peak; RA_Scd_ED: right atrial conduit strain by using the starting points of R-wave peak; RA_Sct_ED: right atrial contractile strain by using the starting points of R-wave peak; RA_Sr_AC: right atrial reservoir strain by using the starting points of P-wave onset; RA_Scd_AC: right atrial conduit strain by using the starting points of P-wave onset; RA_Sct_AC: right atrial contractile strain by using the starting points of P-wave onset; LA_Sr_ED: left atrial reservoir strain by using the starting points of R-wave peak; LA_Scd_ED: left atrial conduit strain by using the starting points of R-wave peak; LA_Sct_ED: left atrial contractile strain by using the starting points of R-wave peak; LA_Sr_AC: left atrial reservoir strain by using the starting points of P-wave onset; LA_Scd_AC: left atrial conduit strain by using the starting points of P-wave onset; LA_Sct_AC: left atrial contractile strain by using the starting points of P-wave onset; *n*: number; %: percentage.

**Table 5:** Diagnostic performance of ventricular and atrial strain parameters in neonatal Ebstein’s anomaly.

Chamber	Strain Parameter	AUC	95% CI	Std. Error ^a^	*p*-Value ^b^	Optimal Cut-Off (%)
Right Ventricle	RV_BRV (%)	0.764	0.634–0.895	0.067	0.001	−22.7
Right Ventricle	RV_MRV (%)	0.653	0.507–0.799	0.074	0.049	−17.2
Right Ventricle	RV_ARV (%)	0.692	0.554–0.830	0.692	0.013	−19.7
Right Ventricle	RV_FWSL (%)	0.703	0.566–0.841	0.070	0.009	−20.85
Right Ventricle	RV_4CSL (%)	0.790	0.674–0.906	0.790	<0.001	−19.2
Left Ventricle	LV_GLS_A4C (%)	0.853	0.753–0.953	0.051	<0.001	−22.05
Left Ventricle	LV_GLS_A2C (%)	0.924	0.861–0.988	0.032	<0.001	−20.25
Left Ventricle	LV_GLS_A3C (%)	0.890	0.810–0.971	0.041	<0.001	−19.35
Left Ventricle	LV_GLS (%)	0.919	0.851–0.986	0.034	<0.001	−21.05
Right Atrium	RA_Sr_ED (%)	0.050	0.000–0.110	0.031	<0.001	-
Right Atrium	RA_Scd_ED (%)	0.907	0.827–0.987	0.041	<0.001	−12.15
Right Atrium	RA_Sct_ED (%)	0.958	0.905–1.000	0.027	<0.001	−13.90
Right Atrium	RA_Sr_AC (%)	0.066	0.000–0.137	0.036	<0.001	-
Right Atrium	RA_Scd_AC (%)	0.811	0.689–0.933	0.062	<0.001	−15.40
Right Atrium	RA_Sct_AC (%)	0.955	0.903–1.000	0.026	<0.001	−9.05
Left Atrium	LA_Sr_ED (%)	0.165	0.054–0.276	0.057	<0.001	-
Left Atrium	LA_Scd_ED (%)	0.802	0.681–0.924	0.062	<0.001	−15.15
Left Atrium	LA_Sct_ED (%)	0.736	0.599–0.872	0.070	0.002	−24.50
Left Atrium	LA_Sr_AC (%)	0.167	0.060–0.275	0.055	<0.001	-
Left Atrium	LA_Scd_AC (%)	0.758	0.626–0.890	0.067	0.001	−15.15
Left Atrium	LA_Sct_AC (%)	0.767	0.641–0.894	0.065	0.001	−14.10

Negative strain values indicate myocardial shortening (more negative = better deformation). AUC: area under the receiver operating characteristic curve; CI: confidence interval. The test result variable(s): has at least one tie between the positive actual state group and the negative actual state group. Statistics may be biased. ^a^. Under the nonparametric assumption; ^b^. Null hypothesis: true area = 0.5. RV_BRV: basal right ventricle segment; RV_MRV: mid right ventricle segment; RV_ARV: apical right ventricle segment; RV_FWSL: right ventricle free wall strain; RV_4CSL: right ventricle apical four-chamber view total strain; LV_GLS_A4C: left ventricular apical four-chamber longitudinal strain; LV_GLS_A2C: left ventricular apical two-chamber longitudinal strain; LV_GLS_A3C: left ventricular apical three-chamber longitudinal strain; LV_GLS: left ventricular global longitudinal strain; RA_Sr_ED: right atrial reservoir strain by using the starting points of R-wave peak; RA_Scd_ED: right atrial conduit strain by using the starting points of R-wave peak; RA_Sct_ED: right atrial contractile strain by using the starting points of R-wave peak; RA_Sr_AC: right atrial reservoir strain by using the starting points of P-wave onset; RA_Scd_AC: right atrial conduit strain by using the starting points of P-wave onset; RA_Sct_AC: right atrial contractile strain by using the starting points of P-wave onset; LA_Sr_ED: left atrial reservoir strain by using the starting points of R-wave peak; LA_Scd_ED: left atrial conduit strain by using the starting points of R-wave peak; LA_Sct_ED: left atrial contractile strain by using the starting points of R-wave peak; LA_Sr_AC: left atrial reservoir strain by using the starting points of P-wave onset; LA_Scd_AC: left atrial conduit strain by using the starting points of P-wave onset; LA_Sct_AC: left atrial contractile strain by using the starting points of P-wave onset; %: percentage.

## Data Availability

The original contributions presented in this study are included in the article. Further inquiries can be directed to the corresponding author.

## Supplementary Materials

The following supporting information can be downloaded at: https://www.mdpi.com/article/10.3390/life16040670/s1, Table S1. Structural and functional echocardiographic parameters by Carpentier type in Ebstein’s anomaly neonates at birth. Table S2. Comparison of the echocardiographic strain indices between control group and the Ebstein’s anomaly neonates according to Carpentier’s classification at birth.

## Abbreviations

The following abbreviations are used in this manuscript:

A	Late diastolic inflow velocity (A wave) obtained by pulsed Doppler
A′	Late diastolic annular velocity (A′ wave) obtained by tissue Doppler echocardiography
AC	Atrial contraction
aRV	Atrialized right ventricle
ASE	American Society of Echocardiography
ASD	Atrial septal defect
AUC	Area under the curve
AVB	Atrioventricular block
AVRT	Atrioventricular re-entry tachycardia
A2C	Apical two-chamber view
A3C	Apical three-chamber view
A4C	Apical four-chamber view
BE	Base excess
BL	Birth length
BP	Blood pressure
BSA	Body surface area
BSA z-score	Body surface area z score
BW	Birth weight
CT index	Cardiothoracic index
E	Early diastolic inflow velocity (E wave) obtained by pulsed Doppler
EA	Ebstein’s anomaly
E′	Early diastolic annular velocity (E′ wave) obtained by tissue Doppler echocardiography
E/A	Ratio of early to late diastolic inflow velocities
E/E′	Ratio of early diastolic inflow velocity to early diastolic annular velocity
ED	End-diastole
EF	Ejection fraction
FiO_2_	Fraction of inspired oxygen
FRV	Functional right ventricle
GLS	Global longitudinal strain
GOSE	Great Ormond Street Echocardiography Score
HR	Heart rate
HR z-score	Heart rate z score
IVCT	Isovolumic contraction time
IVRT	Isovolumic relaxation time
LA	Left atrium
LASr	Left atrial reservoir strain
LAScd	Left atrial conduit strain
LASct	Left atrial contractile strain
LA_Sr_ED	Left atrial reservoir strain using R-wave peak
LA_Scd_ED	Left atrial conduit strain using R-wave peak
LA_Sct_ED	Left atrial contractile strain using R-wave peak
LA_Sr_AC	Left atrial reservoir strain using P-wave onset
LA_Scd_AC	Left atrial conduit strain using P-wave onset
LA_Sct_AC	Left atrial contractile strain using P-wave onset
LV	Left ventricle
LV_EF	Left ventricular ejection fraction
LV_GLS	Left ventricular global longitudinal strain
LV_BIS	Left ventricle basal inferoseptal segment
LV_MIS	Left ventricle mid inferoseptal segment
LV_AIS	Left ventricle apical inferoseptal segment
LV_BAL	Left ventricle basal anterolateral segment
LV_MAL	Left ventricle mid anterolateral segment
LV_AAL	Left ventricle apical anterolateral segment
LV_BI	Left ventricle basal inferior segment
LV_MI	Left ventricle mid inferior segment
LV_AI	Left ventricle apical inferior segment
LV_BA	Left ventricle basal anterior segment
LV_MA	Left ventricle mid anterior segment
LV_AA	Left ventricle apical anterior segment (A2C)
LV_BIL	Left ventricle basal inferolateral segment
LV_MIL	Left ventricle mid inferolateral segment
LV_AL	Left ventricle apical lateral segment
LV_BAS	Left ventricle basal anteroseptal segment
LV_MAS	Left ventricle mid anteroseptal segment
LV_AA2	Left ventricle apical anterior segment (A3C)
LV_GLS_A4C	LV apical four-chamber longitudinal strain
LV_GLS_A2C	LV apical two-chamber longitudinal strain
LV_GLS_A3C	LV apical three-chamber longitudinal strain
MAPSE	Mitral annular plane systolic excursion
mBP	Mean blood pressure
mBP z-score	Mean blood pressure z score
Mitral_A′	Mitral annular late diastolic velocity (A′ wave)
Mitral_E′	Mitral annular early diastolic velocity (E′ wave)
Mitral_E/A	Ratio of early to late mitral inflow velocity
Mitral_E/E′	Ratio of early mitral inflow velocity to E′
Mitral_IVCT	LV isovolumic contraction time
Mitral_IVRT	LV isovolumic relaxation time
Mitral_S	Mitral annular systolic velocity
NO	Nitric oxide
NT-proBNP	N-terminal pro–B-type natriuretic peptide
O_2_	Oxygen
pCO_2_	Partial pressure of carbon dioxide
PDA	Patent ductus arteriosus
pH	Acidity or alkalinity of blood
PGE_1_	Prostaglandin E1
pO_2_	Partial pressure of oxygen
PR	Pulmonary regurgitation
PV	Pulmonary valve
PVR	Pulmonary vascular resistance
RA	Right atrium
RASr	Right atrial reservoir strain
RAScd	Right atrial conduit strain
RASct	Right atrial contractile strain
RA_Scd_ED	RA conduit strain using R-wave peak
RA_Sct_ED	RA contractile strain using R-wave peak
RA_Sr_AC	RA reservoir strain using P-wave onset
RA_Scd_AC	RA conduit strain using P-wave onset
RA_Sct_AC	RA contractile strain using P-wave onset
ROC	Receiver operating characteristic
ROI	Region of interest
RV	Right ventricle
RVFF	Right ventricular forward flow
RV_ARV	RV apical segment
RV_BRV	RV basal segment
RV_FAC	Right ventricular fractional area change
RV_FWSL	RV free-wall longitudinal strain
RV_4CSL	RV four-chamber longitudinal strain
RV_MRV	RV mid segment
S	Annular systolic velocity (S wave)
SaO_2_	Peripheral oxygen saturation
SD	Standard deviation
SO_2_	Oxygen saturation
STE	Speckle-tracking echocardiography
SVT	Supraventricular tachycardia
TAPSE	Tricuspid annular plane systolic excursion
TR	Tricuspid regurgitation
Tricuspid_A′	Tricuspid annular late diastolic velocity
Tricuspid_E′	Tricuspid annular early diastolic velocity
Tricuspid_E/A	Ratio of early to late tricuspid inflow velocity
Tricuspid_E/E′	Ratio of early tricuspid inflow velocity to E′
Tricuspid_IVCT	RV isovolumic contraction time
Tricuspid_IVRT	RV isovolumic relaxation time
Tricuspid_S	Tricuspid annular systolic velocity
TV	Tricuspid valve
VSD	Ventricular septal defect
WPW	Wolff–Parkinson–White syndrome

## References

[1] Kumar T.K.S. (2021). Ebstein’s anomaly in the neonate. Indian J. Thorac. Cardiovasc. Surg..

[2] Neumann S., Rüffer A., Sachweh J., Biermann D., Herrmann J., Jerosch-Herold M., Hazekamp M., Sinning C., Zengin E., Blankenberg S. (2021). Narrative review of Ebstein’s anomaly beyond childhood: Imaging, surgery, and future perspectives. Cardiovasc. Diagn. Ther..

[3] Acar P., Abadir S., Roux D., Taktak A., Dulac Y., Glock Y., Fournial G. (2006). Ebstein’s anomaly assessed by real-time 3-D echocardiography. Ann. Thorac. Surg..

[4] Mah K., Mertens L. (2022). Echocardiographic assessment of right ventricular function in pediatric heart disease: A practical clinical approach. CJC Pediatr. Congenit. Heart Dis..

[5] Ricci P., Ostenfeld E., Flick C., West C., Babu-Narayan S., Nihoyannopoulos P., Li W. (2020). Assessment of biventricular function using advanced echocardiography in patients with Ebstein’s anomaly pre- and post-tricuspid valve surgery. Eur. Heart J. Cardiovasc. Imaging.

[6] Prota C., Di Salvo G., Sabatino J., Josen M., Paredes J., Sirico D., Uy Pernia M., Hoschtitzky A., Michielon G., Citro R. (2019). Prognostic value of echocardiographic parameters in pediatric patients with Ebstein’s anomaly. Int. J. Cardiol..

[7] Dorobantu D.M., Amir N.H., Wadey C.A., Sharma C., Stuart A.G., Williams C.A., Pieles G.E. (2024). The role of speckle-tracking echocardiography in congenital heart disease: A systematic review and meta-analysis. J. Am. Soc. Echocardiogr..

[8] Lai W.W., Geva T., Shirali G., Frommelt P.C., Humes R.A., Brook M.M., Pignatelli R.H., Rychik J. (2006). Guidelines and standards for performance of a pediatric echocardiogram: A report from the task force of the Pediatric Council of the American Society of Echocardiography. J. Am. Soc. Echocardiogr..

[9] Engle M.A., Payne T.P.B., Bruins C., Taussig H.B. (1950). Ebstein’s anomaly of the tricuspid valve; report of three cases and analysis of the clinical syndrome. Circulation.

[10] Moura C., Guimarães H., Areias J.C., Moreira J. (2001). Ebstein’s anomaly in neonates. Rev. Port. Cardiol..

[11] Modi D., Dasara D., Singha N., Shinde K. (2025). Ebstein’s anomaly: Glimpses into pathophysiology, prevalence, diagnostic updates, and treatment strategies through evolution. Multidiscip. Rev..

[12] Sainathan S., da Fonseca da Silva L., Jose Pedro da Silva J.P. (2020). Ebstein’s anomaly: Contemporary management strategies. J. Thorac. Dis..

[13] Pasqualin G., Ciconte G., Boccellino A., Chessa M., Marcolin C., Micaglio E., Pappone C., Sturla F., Giamberti A. (2024). Ebstein’s anomaly in children and adults: Multidisciplinary insights into imaging and therapy. Heart.

[14] Celermajer D.S., Cullen S., Sullivan I.D., Spiegelhalter D.J., Wyse R.K.H., Deanfield J.E. (1992). Outcome in neonates with Ebstein’s anomaly. J. Am. Coll. Cardiol..

[15] Celermajer D.S., Dodd S.M.D., Greenwald S.E., Wyse R.K., Deanfield J.E. (1992). Morbid anatomy in neonates with Ebstein’s anomaly of the tricuspid valve: Pathophysiologic and clinical implications. J. Am. Coll. Cardiol..

[16] Starnes V.A., Pitlick P.T., Bernstein D., Griffin M.L., Choy M., Shumway N.E. (1991). Ebstein’s anomaly appearing in the neonate. A new surgical approach. J. Thorac. Cardiovasc. Surg..

[17] Yu J.J., Yun T.-J., Won H.-S., Im Y.M., Lee B.S., Kang S.Y., Ko H.K., Park C.S., Park J.-J., Gwak M. (2013). Outcome of neonates with Ebstein’s anomaly in the current era. Pediatr. Cardiol..

[18] Holst K.A., Connolly H.M., Dearani J.A. (2019). Ebstein’s anomaly. Methodist DeBakey Cardiovasc. J..

[19] Freud L.R., McElhinney D.B., Kalish B.T., Escobar-Diaz M.C., Komarlu R., Puchalski M.D., Jaeggi E.T., Szwast A.L., Freire G., Levasseur S.M. (2020). Risk factors for mortality and circulatory outcome among neonates prenatally diagnosed with Ebstein anomaly or tricuspid valve dysplasia: A multicenter study. J. Am. Heart Assoc..

[20] Correra A., Mauriello A., Di Peppo M., D’Andrea A., Russo V., Esposito G., Brunetti N.D. (2025). Atrial Septal Defect and Heart Rhythm Disorders: Physiopathological Linkage and Clinical Perspectives. Biomedicines.

[21] Adıgüzel A., Aypar E., Karagöz T., Ertuğrul I., Aykan H.H., Alehan D., Güvener M., Yılmaz M., Demircin M., Doğan R. (2025). Ebstein’s anomaly in children and young adults: Clinical features, arrhythmia, surgical management, and factors affecting arrhythmia and mortality. Cardiol. Young.

[22] Singh D.P., Hussain K., Horenstein M.S., Mahajan K. (2024). Ebstein’s anomaly. StatPearls.

[23] Teramachi Y., Hornberger L.K., Howley L., van der Velde M.E., Eckersley L.G. (2022). Left Ventricular Dysfunction in Neonatal Ebstein’s Anomaly and Tricuspid Valve Dysplasia. J. Am. Soc. Echocardiogr..

[24] Levy P.T., Machefsky A., Sanchez A.A., Patel M.D., Rogal S., Fowler S., Singh G.K. (2016). Reference Ranges of Left Ventricular Strain Measures by Two-Dimensional Speckle-Tracking Echocardiography in Children: A Systematic Review and Meta-Analysis. J. Am. Soc. Echocardiogr..

[25] Friedberg M.K., Mertens L. (2017). Deformation Imaging in Selected Congenital Heart Disease: Are We There Yet?. J. Am. Soc. Echocardiogr..

[26] Kutty S., Li L., Hasan R., Danford D.A. (2021). Atrial Mechanics and Function in Congenital Heart Disease Assessed by Speckle-Tracking Echocardiography: A Systematic Review. J. Am. Soc. Echocardiogr..

