# Supplementary material for: Advanced Echocardiographic Characterization of Neonatal Ebstein’s Anomaly Using Myocardial Deformation Imaging: A Single-Center Study
Source: Life (Basel). 2026 Apr 14;16(4):670. doi: 10.3390/life16040670 (PMC13117587; doi:10.3390/life16040670)
Supplement: Supplementary file 1 [file life-16-00670-s001.zip › life-4162899-supplementary.pdf]

Table S1. Structural and functional echocardiographic parameters by Carpentier type in Ebstein`s anomaly neonates at birth

Variable	EA group Carpentier A (n=8)	EA group Carpentier B (n=5)	EA group Carpentier C (n=3)	p-value
Structural echocardiographic parameters				
RA area (cm²)	4.92 ± 2.22 (2.6–9.3)	7.03 ± 1.13 (5.9–8.8)	6.63 ± 2.54 (4.4–9.4)	0.077*
aRV area (cm²)	3.06 ± 1.41 (1.9–6.1)	3.06 ± 1.15 (1.1–4.0)	1.86 ± 1.01 (0.9–2.9)	-
FRV area (cm²)	2.01 ± 0.56 (1.2–3.0)	2.52 ± 0.99 (1.6–3.8)	2.38 ± 0.33 2.1–2.7)	0.157*
LA area (cm²)	3.10 ± 1.13 (1.6–5.1)	3.35 ± 0.83 (2.5–4.6)	3.68 ± 0.77 2.8–4.2)	0.371*
LV area (cm²)	0.62 ± 0.20 (0.4–0.9)	0.75 ± 0.15 (0.6–0.9)	0.90 ± 0.45 (0.5–1.4)	0.611*
RV index	13.7 ± 5.6 (9.0–25.4)	13.9 ± 5.2 (4.9–18.3)	9.36 ± 5.16 (3.7–13.8)	0.346*
LV index	13.9 ± 4.3 (7–21.3)	15.4 ± 4.1 (10.3–20.7)	17.5 ± 2.2 (15.6–19.9)	0.074**
RA index	**21.8 ± 8.5 (12.8–37.4)**	**32.1 ± 5.2 (26.7–38.5)**	**32.8 ± 16.9 (21–52.2)**	0.026**
GOSE index	—	—	—	0.392
RV-RA gradient (mmHg)	44.8 ± 9.6 (30–60)	46.6 ± 10.9 (37–61)	32.7 ± 14.5 (16–42)	0.157**
TV annulus (mm)	14.7 ± 2.1 (11.4–17.4)	15.6 ± 2.4 (13.2–19)	13.8 ± 1.7 (12.1–15.5)	0.570*
TV annulus z-score	2.17 ± 1.15 (0.46–3.4)	2.40 ± 1.53 (0.3–4.17)	1.22 ± 1.07 (−0.01–1.87)	0.417*
PV annulus (mm)	7.56 ± 0.84 (6.5–9.4)	7.44 ± 1.05 (6.3–9.1)	4.62 ± 3.45 (0.7–7)	0.075*
PV annulus z-score	−0.62 ± 0.95 (−1.35–1.54)	−0.98 ± 0.60 (−1.53–−0.14)	−1.35 ± 0.50 (−1.9–−0.96)	0.442*
Functional echocardiographic parameters				
TAPSE (mm)	9.40 ± 1.7 (7.1–12.4)	9.12 ± 1.7 (7.1–11.6)	5.73 ± 1.4 (4.5–7.2)	0.056* (C–A, 0.052)
RV_FAC (%)	**42.4 ± 5.4 (32.9–48.7)**	**37.8 ± 3.4 (34.5–42.5)**	**27.7 ± 11.7 (15–38)**	0.044** (C–A, 0.051)
Tricuspid_E/A	0.90 ± 0.11 (0.77–1.10)	0.82 ± 0.06 (0.71–0.87)	0.68 ± 0.14 (0.6–0.84)	0.070*
Tricuspid_E′ (cm/s)	10.34 ± 3.5 (4.7–16.2)	10.56 ± 2.7 (8.2–14.9)	7.87 ± 2.1 (5.5–9.6)	0.302*
Tricuspid_A′ (cm/s)	10.44 ± 2.7 (5.9–12.7)	11.56 ± 1.3 (10.5–13.7)	7.77 ± 1.6 (6.5–9.6)	0.132*
Tricuspid_S (cm/s)	**5.80 ± 0.7 (5.0–6.9)**	**5.73 ± 1.5 (4.8–8.4)**	**4.17 ± 0.9 (3.2–4.9)**	0.034* (C–A, 0.028)
Tricuspid_E/E′	0.06 ± 0.02	0.05 ± 0.01	0.06 ± 0.01	0.941*

	(0.04–0.10)	(0.04–0.07)	(0.05–0.07)	
Tricuspid_IVCT (ms)	57.5 ± 13.4<br>(34–75)	67.2 ± 16.1<br>(45–88)	78.0 ± 6.1<br>(74–85)	0.074**
Tricuspid_IVRT (ms)	**66.5 ± 17.1<br>(45–100)**	**79.4 ± 9.6<br>(66–89)**	**81.0 ± 5.2<br>(78–87)**	0.023**<br>(A–C, 0.025)
LV_EF (%)	53.1 ± 3.6<br>(48–60)	50.8 ± 7.2<br>(40–60)	38.1 ± 13.5<br>(25–52)	0.150*
MAPSE (mm)	8.21 ± 1.5<br>(6.1–10.5)	7.94 ± 1.4<br>(5.9–9.2)	5.43 ± 2.6<br>(3.4–8.4)	0.269*
Mitral_E/A	0.98 ± 0.20<br>(0.65–1.28)	0.91 ± 0.18<br>(0.71–1.16)	0.77 ± 0.15<br>(0.6–0.9)	0.306*
Mitral_E′ (cm/s)	12.1 ± 3.9<br>(6.4–18.5)	13.3 ± 4.3<br>(8.7–19.6)	7.77 ± 1.4<br>(6.2–8.9)	0.077*
Mitral_A′ (cm/s)	11.5 ± 2.7<br>(6.6–14.9)	11.1 ± 2.6<br>(8.6–14.2)	7.77 ± 1.5<br>(6.7–9.5)	0.167*
Mitral_S (cm/s)	5.61 ± 0.5<br>(4.7–6.3)	5.80 ± 1.6<br>(4.8–8.6)	4.30 ± 1.2<br>(3.0–5.3)	0.133*
Mitral_E/E′	0.05 ± 0.02<br>(0.04–0.09)	0.04 ± 0.01<br>(0.03–0.05)	0.05 ± 0.02<br>(0.04–0.07)	0.161*
Mitral_IVCT (ms)	58.0 ± 10.4<br>(37–70)	65.2 ± 17.9<br>(40–90)	84.7 ± 4.2<br>(80–88)	0.051*<br>(A–C, 0.046)
Mitral_IVRT (ms)	**62.5 ± 9.3<br>(48–75)**	**70.2 ± 18.3<br>(40–89)**	**86.7 ± 4.2<br>(82–90)**	0.029*<br>(A–C, 0.025)

Data are expressed as mean ± SD (range). Significance tested by Mann–Whitney U test; $p<0.05$ considered significant.

*Independent-Samples Kruskal–Wallis Test; **Independent-Samples Median Test.

EA: Ebstein`s anomaly; Carpentier classification of Ebstein`s anomaly: type A, type B, type C; RA: right atrium; RV: right ventricle; aRV: atrialized right ventricle; FRV: functional right ventricle; LA: left atrium; LV: left ventricle; GOSE: Great Ormond Street Echocardiography Score; TV: tricuspid valve; PV: pulmonary valve; TAPSE: tricuspid annular plain systolic excursion; RV_FAC: right ventricle fractional change area; tricuspid_E/A; the ratio between early tricuspid inflow velocity and late tricuspid inflow velocity; tricuspid_E`: tricuspid annular early diastolic velocity (E`wave) obtained by tissue Doppler echocardiography; tricuspid_A`: tricuspid annular late diastolic velocity (A`wave) obtained by tissue Doppler echocardiography; tricuspid_S: tricuspid annular systolic velocity (S wave) obtained by tissue Doppler echocardiography: tricuspid E/E`: the ratio between early tricuspid inflow velocity and tricuspid annular early diastolic velocity; tricuspid_IVCT: right ventricle isovolumic contraction time; tricuspid_IVRT: right ventricle isovolumic relaxation time; LV_EF: left ventricle ejection fraction; MAPSE: mitral annular plain systolic excursion, mitral_E/A: the ratio between early mitral inflow velocity and late mitral inflow velocity; mitral_E`: mitral annular early diastolic velocity (E`wave) obtained by tissue Doppler echocardiography; mitral_A`: mitral annular late diastolic velocity (A`wave) obtained by tissue Doppler echocardiography; mitral_S: mitral annular systolic velocity (S wave) obtained by tissue Doppler echocardiography; mitral_E/E`: the ratio between early mitral inflow velocity and mitral annular early diastolic velocity (E/E'); mitral_IVCT: left ventricle

isovolumic contraction time; mitral_IVRT: left ventricle isovolumic relaxation time; n:number.

Table S2. Comparison of the echocardiographic strain indices between control group and the Ebstein`s anomaly neonates according to Carpentier`s classification at birth

Variables	Control group (n=26)	EA group Carpentier A (n=8)	EA group Carpentier B (n=5)	EA group Carpentier C (n=3)	p-value
Right ventricle segmental strain					
RV_BRV (%)	-30.5 ± 6.0 (-38.9 / -16.8)	-19.8 ± 9.0 (-35.0 / -10.3)	-17.5 ± 6.7 (-24.2 / -9.0)	-11.3 ± 8.0 (-16.8 / -2.1)	<0.001*[1,2,3]
RV_MRV (%)	-24.8 ± 4.7 (-33.5 / -17.3)	-18.7 ± 7.4 (-32.1 / -11.2)	-15.7 ± 7.1 (-23.7 / -8.1)	-11.0 ± 10.2 (-21.5 / -1.1)	0.006*
RV_ARV (%)	-24.6 ± 4.1 (-35.5 / -17.5)	-18.3 ± 6.2 (-28.1 / -10.6)	-18.5 ± 8.8 (-28.0 / -7.7)	-11.8 ± 7.6 (-18.4 / -3.5)	0.007*[3]
RV_RVFWSL (%)	-26.6 ± 4.4 (-35.8 / -18.5)	-19.3 ± 7.7 (-32.8 / -11.4)	-17.6 ± 7.4 (-25.5 / -9.2)	-11.7 ± 7.8 (-17.5 / -2.8)	0.003*[3]
RV_RV4CSL (%)	-23.9 ± 2.9 (-28.6 / -18.1)	-16.5 ± 4.0 (-22.4 / -11.9)	-17.4 ± 6.1 (-23.9 / -9.7)	-10.1 ± 6.5 (-14.8 / -2.7)	<0.001*[2,3]
Left Ventricle Segmental Strain					
LV_BIS (%)	-18.2 ± 3.9 (-27.3 / -8.8)	-11.3 ± 2.8 (-14.5 / -7.0)	-9.5 ± 6.1 (-18.9 / -2.8)	-7.5 ± 9.1 (-17.7 / -0.5)	<0.001*[1,3]
LV_MIS (%)	-21.6 ± 4.4 (-32.4 / -13.6)	-15.3 ± 4.2 (-20.7 / -7.6)	-15.2 ± 5.6 (-22.7 / -9.1)	-13.6 ± 7.9 (-20.8 / -5.2)	0.002*[1]
LV_AIS (%)	-25.1 ± 4.7 (-35.1 / -13.4)	-21.5 ± 4.7 (-27.3 / -15.4)	-20.5 ± 3.5 (-26.0 / -16.8)	-18.2 ± 12.4 (-30.4 / -5.7)	0.107*
LV_BAL (%)	-26.8 ± 7.1 (-42.2 / -14.2)	-18.4 ± 9.0 (-36.6 / -10.2)	-13.2 ± 7.4 (-21.5 / -1.8)	-11.1 ± 9.6 (-20.7 / -1.5)	<0.001*[2]
LV_MAL (%)	-18.7 ± 2.9 (-25.9 / -11.0)	-10.9 ± 3.4 (-15.8 / -4.9)	-11.9 ± 6.4 (-17.9 / -5.2)	-9.8 ± 5.7 (-14.7 / -3.5)	<0.001*[1,3]
LV_AAL (%)	-22.7 ± 5.3 (-33.4 / -15.5)	-19.4 ± 7.3 (-25.7 / -7.5)	-13.1 ± 8.7 (-23.1 / -2.2)	-8.5 ± 9.9 (-19.9 / -2.6)	0.037*
LV_BI (%)	-20.1 ± 4.1 (-29.6 / -9.6)	-15.0 ± 6.7 (-27.4 / -6.1)	-12.0 ± 4.8 (-18.1 / -5.8)	-8.7 ± 8.1 (-18.0 / -3.2)	0.002*[2]
LV_MI (%)	-20.6 ± 4.5 (-26.4 / -9.6)	-14.1 ± 2.2 (-16.9 / -9.9)	-13.3 ± 4.7 (-18.9 / -6.0)	-12.2 ± 5.8 (-16.2 / -5.5)	<0.001*[1,2]
LV_AI (%)	-24.6 ± 3.9 (-30.8 / -16.0)	-18.8 ± 6.7 (-24.8 / -3.6)	-17.1 ± 6.2 (-24.5 / -7.4)	-10.2 ± 8.0 (-18.1 / -2.1)	0.001*[3]
LV_BA (%)	-21.8 ± 4.4 (-30.2 / -14.0)	-15.9 ± 5.3 (-21.3 / -8.0)	-13.4 ± 4.2 (-17.5 / -7.0)	-7.5 ± 3.6 (-9.9 / -3.4)	<0.001*[2,3]
LV_MA (%)	-21.2 ± 3.5 (-28.8 / -13.7)	-12.0 ± 3.0 (-16.5 / -8.0)	-14.3 ± 5.3 (-22.2 / -8.4)	-8.7 ± 5.6 (-12.5 / -2.2)	<0.001*[1,3]
LV_AA (%)	-22.9 ± 9.9 (-32.3 / 18.6)	-17.8 ± 4.4 (-22.1 / -9.3)	-15.2 ± 5.7 (-23.6 / -10.4)	-13.9 ± 11.0 (-23.5 / -1.9)	0.017*
LV_BIL (%)	-23.4 ± 6.1	-19.3 ± 11.1	-15.2 ± 4.2	-7.2 ± 7.1	0.004*[3]

	(-39.4 / -13.4)	(-36.2 / -5.4)	(-21.6 / -11.2)	(-15.4 / -2.8)	
LV_MIL (%)	-20.8 ± 5.8<br>(-30.2 / -10.7)	-10.3 ± 3.3<br>(-14.6 / -4.0)	-14.5 ± 3.6<br>(-19.5 / -10.4)	-6.4 ± 4.5<br>(-10.8 / -1.8)	<0.001*[1,3]
LV_AL (%)	-28.7 ± 30.3<br>(-175.0 / -14.9)	-16.7 ± 3.5<br>(-22.6 / -11.4)	-17.1 ± 2.5<br>(-19.3 / -13.2)	-15.2 ± 10.8<br>(-25.6 / -4.0)	0.007*[1]
LV_BAS (%)	-20.2 ± 3.5<br>(-30.2 / -13.3)	-11.2 ± 5.7<br>(-22.2 / -3.0)	-14.8 ± 4.1<br>(-22.2 / -12.5)	-7.5 ± 7.5<br>(-16.2 / -2.6)	<0.001*[1,3]
LV_MAS (%)	-21.0 ± 3.9<br>(-28.4 / -12.9)	-14.9 ± 4.1<br>(-21.8 / -9.7)	-15.3 ± 4.5<br>(-20.9 / -9.8)	-10.4 ± 6.9<br>(-17.3 / -3.6)	<0.001*[1]
LV_AA[2] (%)	-22.1 ± 5.0<br>(-33.2 / -15.4)	-24.9 ± 4.8<br>(-36.1 / -20.2)	-21.1 ± 9.3<br>(-35.5 / -10.0)	-14.7 ± 11.6<br>(-23.3 / -1.5)	0.171*
LV_GLS_A4C (%)	-22.2 ± 2.3<br>(-26.6 / -18.9)	-16.2 ± 2.6<br>(-19.4 / -12.4)	-13.5 ± 2.9<br>(-17.5 / -9.5)	-10.8 ± 6.3<br>(-15.5 / -3.6)	<0.001*[1,2,3]
LV_GLS_A2C (%)	-22.1 ± 2.1<br>(-25.7 / -18.6)	-15.6 ± 2.1<br>(-17.8 / -12.4)	-14.0 ± 3.8<br>(-19.3 / -9.0)	-10.8 ± 6.7<br>(-15.5 / -3.0)	<0.001*[1,2,3]
LV_GLS_A3C (%)	-21.8 ± 2.5<br>(-26.6 / -18.0)	-16.1 ± 1.7<br>(-18.4 / -13.4)	-16.4 ± 2.2<br>(-18.4 / -13.2)	-10.2 ± 6.9<br>(-16.2 / -2.7)	<0.001*[1,2,3]
LV_GLS (%)	-22.3 ± 1.9<br>(-25.6 / -19.3)	-16.0 ± 1.9<br>(-18.4 / -13.6)	-14.6 ± 2.3<br>(-18.3 / -12.4)	-10.5 ± 6.4<br>(-14.3 / -3.1)	<0.001*[1,2,3]
Right atrium strain analysis					
RA_Sr_ED (%)	36.9 ± 8.9<br>(16.6 / 58.5)	12.9 ± 5.9<br>(2.1 / 23.2)	14.0 ± 2.8<br>(10.7 / 17.5)	8.8 ± 6.0<br>(2.0 / 13.4)	<0.001*[1,2,3]
RA_Scd_ED (%)	-20.1 ± 5.5<br>(-27.7 / -6.5)	-8.1 ± 3.7<br>(-14.8 / -2.2)	-7.1 ± 3.2<br>(-12.7 / -4.7)	-5.8 ± 3.9<br>(-9.5 / -1.8)	<0.001*[1,2,3]
RA_Sct_ED (%)	-19.2 ± 6.1<br>(-37.4 / -6.4)	-4.7 ± 2.9<br>(-8.2 / 0.2)	-6.7 ± 2.2<br>(-10.2 / -4.6)	-2.4 ± 1.7<br>(-3.9 / -0.5)	<0.001*[1,2,3]
RA_Sr_AC (%)	34.1 ± 8.9<br>(15.7 / 53.6)	13.4 ± 6.1<br>(2.1 / 23.5)	13.4 ± 2.4<br>(10.6 / 15.8)	8.6 ± 5.7<br>(2.2 / 13.0)	<0.001*[1,2,3]
RA_Scd_AC (%)	-16.5 ± 9.0<br>(-25.4 / 19.6)	-10.0 ± 5.9<br>(-22.1 / -2.2)	-5.1 ± 6.1<br>(-12.4 / 4.5)	-5.7 ± 3.7<br>(-9.2 / -1.9)	<0.001*[2]
RA_Sct_AC (%)	-17.4 ± 5.1<br>(-27.2 / -6.1)	-4.0 ± 2.4<br>(-6.7 / -0.2)	-2.5 ± 6.4<br>(-6.7 / 8.8)	-2.3 ± 1.7<br>(-3.8 / -0.5)	<0.001*[1,2,3]
Left atrium strain analysis					
LA_Sr_ED (%)	37.5 ± 8.2<br>(26.4 / 63.0)	23.2 ± 9.7<br>(11.6 / 40.7)	11.5 ± 17.4<br>(-19.5 / 20.8)	20.8 ± 10.5<br>(9.6 / 30.4)	<0.001*[1,2]
LA_Scd_ED (%)	-23.0 ± 4.3<br>(-29.8 / -14.4)	-12.7 ± 4.0<br>(-17.3 / -6.4)	-13.0 ± 6.1<br>(-19.5 / -5.7)	-12.2 ± 8.8<br>(-18.8 / -2.2)	<0.001*[1,2]
LA_Sct_ED (%)	-17.2 ± 7.6<br>(-35.8 / -2.6)	-10.6 ± 6.6<br>(-23.4 / -2.6)	-8.9 ± 4.1<br>(-14.2 / -4.9)	-6.7 ± 5.5<br>(-11.6 / -0.8)	0.014*
LA_Sr_AC (%)	34.3 ± 6.9<br>(23.7 / 46.6)	21.3 ± 7.0<br>(11.3 / 33.1)	18.2 ± 1.5<br>(16.7 / 19.9)	19.8 ± 9.3<br>(9.4 / 27.4)	<0.001*[1,2]
LA_Scd_AC (%)	-21.1 ± 4.4<br>(-25.8 / -12.0)	-13.8 ± 4.6<br>(-23.7 / -8.8)	-12.5 ± 6.4<br>19.2 / -5.0)	-11.5 ± 8.2<br>(-16.9 / -2.1)	<0.001*[1]
LA_Sct_AC (%)	-15.6 ± 6.5<br>(-26.4 / -2.5)	-8.8 ± 5.5<br>(-19.0 / -2.6)	-6.8 ± 4.8<br>(-12.4 / -0.4)	-6.3 ± 5.0<br>(-10.5 / -0.7)	0.005*

Negative strain values indicate myocardial shortening (more negative=better deformation).

Data are expressed as mean ± SD (range). Significance tested by Mann–Whitney U test; $p<0.05$ considered significant.

*Independent-Samples Kruskal–Wallis Test, ** pairwise comparisons with Bonferroni test (adjusted sig.)

1:A-control; 2: B-control; 3:C-control.

EA:Ebstein`s anomaly; RV_BRV: basal right ventricle segment; RV_MRV: mid right ventricle segment; RV_ARV: apical right ventricle segment; RV_FWSL: right ventricle free wall strain; RV_4CSL: right ventricle apical four-chamber view total strain; LV_BIS: left ventricle basal inferoseptal segment; LV_MIS: left ventricle mid inferoseptal segment; LV_AIS: left ventricle apical inferoseptal segment; LV_BAL: left ventricle basal anterolateral segment; LV_MAL: left ventricle mid anterolateral segment; LV_AAL: left ventricle apical anterolateral segment; LV_BI: left ventricle basal inferior segment; LV_MI: left ventricle mid inferior segment; LV_AI: left ventricle apical inferior segment; LV_BA:left ventricle basal anterior segment; LV_MA: left ventricle mid anterior segment; LV_AA: left ventricle apical anterior segment (assessed from apical two-chamber view); LV_BIL: left ventricle basal inferolateral segment; LV_MIL: left ventricle mid inferolateral segment; LV_AL: left ventricle apical lateral segment; LV_BAS: left ventricle basal anteroseptal segment; LV_MAS: left ventricle mid anteroseptal segment; LV_AA2:left ventricle apical anterior segment (assessed from apical three-chamber view); LV_GLS_A4C:left ventricular apical four-chamber longitudinal strain; LV_GLS_A2C:left ventricular apical two-chamber longitudinal strain; LV_GLS_A3C:left ventricular apical three-chamber longitudinal strain; LV_GLS: left ventricular global longitudinal strain; RA_Sr_ED: right atrial reservoir strain by using the starting points of R-wave peak; RA_Scd_ED: right atrial conduit strain by using the starting points of R-wave peak; RA_Sct_ED: right atrial contractile strain by using the starting points of R-wave peak; RA_Sr_AC: right atrial reservoir strain by using the starting points of P-wave onset; RA_Scd_AC: right atrial conduit strain by using the starting points of P-wave onset; RA_Sct_AC: right atrial contractile strain by using the starting points of P-wave onset; LA_Sr_ED: left atrial reservoir strain by using the starting points of R-wave peak; LA_Scd_ED: left atrial conduit strain by using the starting points of R-wave peak; LA_Sct_ED: left atrial contractile strain by using the starting points of R-wave peak; LA_Sr_AC: left atrial reservoir strain by using the starting points of P-wave onset; LA_Scd_AC: left atrial conduit strain by using the starting points of P-wave onset; LA_Sct_AC: left atrial contractile strain by using the starting points of P-wave onset; n:number; %:percentage.
